# Clinical Efficacy and Safety of Proton and Carbon Ion Radiotherapy for Prostate Cancer: A Systematic Review and Meta-Analysis

**DOI:** 10.3389/fonc.2021.709530

**Published:** 2021-10-12

**Authors:** Meixuan Li, Xiuxia Li, Liang Yao, Xue Han, Wenlong Yan, Yujun Liu, Yiwen Fu, Yakun Wang, Min Huang, Qiuning Zhang, Xiaohu Wang, Kehu Yang

**Affiliations:** ^1^ Evidence-Based Medicine Center, School of Basic Medical Sciences, Lanzhou University, Lanzhou, China; ^2^ Evidence-Based Social Science Research Center, School of Public Health, Lanzhou University, Lanzhou, China; ^3^ Key Laboratory of Evidence-Based Medicine and Knowledge Translation of Gansu Province, Lanzhou, China; ^4^ Health Technology Assessment Center of Lanzhou University, School of Public Health, Lanzhou University, Lanzhou, China; ^5^ Department of Health Research Methods, Evidence and Impact, McMaster University, Hamilton, ON, Canada; ^6^ The Second School of Clinical Medicine, Lanzhou University, Lanzhou, China; ^7^ The First School of Clinical Medicine, Lanzhou University, Lanzhou, China; ^8^ Institute of Modern Physics, Chinese Academy of Sciences, Lanzhou, China; ^9^ Lanzhou Heavy Ions Hospital, Lanzhou, China

**Keywords:** proton beam therapy, carbon ion radiotherapy, prostate cancer, efficacy, safety, meta-analysis

## Abstract

**Background:**

Carbon ion radiotherapy (CIRT) and proton beam therapy (PBT) are promising methods for prostate cancer, however, the consensus of an increasing number of studies has not been reached. We aimed to provide systematic evidence for evaluating the efficacy and safety of CIRT and PBT for prostate cancer by comparing photon radiotherapy.

**Materials and Methods:**

We searched for studies focusing on CIRT and PBT for prostate cancer in four online databases until July 2021. Two independent reviewers assessed the quality of included studies and used the GRADE approach to rate the quality of evidence. R 4.0.2 software was used to conduct the meta-analysis. A meta-regression test was performed based on the study design and tumor stage of each study.

**Results:**

A total of 33 studies including 13 CIRT- and 20 PBT-related publications, involving 54,101, participants were included. The quality of the included studies was found to be either low or moderate quality. Random model single-arm meta-analysis showed that both the CIRT and PBT have favorable efficacy and safety, with similar 5-year overall survival (OS) (94 *vs* 92%), the incidence of grade 2 or greater acute genitourinary (AGU) toxicity (5 *vs* 13%), late genitourinary (LGU) toxicity (4 *vs* 5%), acute gastrointestinal (AGI) toxicity (1 *vs* 1%), and late gastrointestinal (LGI) toxicity (2 *vs* 4%). However, compared with CIRT and PBT, photon radiotherapy was associated with lower 5-year OS (72–73%) and a higher incidence of grade 2 or greater AGU (28–29%), LGU (13–14%), AGI (14–19%), and LGI toxicity (8–10%). The meta-analysis showed the 3-, 4-, and 5-year local control rate (LCR) of CIRT for prostate cancer was 98, 97, and 99%; the 3-, 4-, 5-, and 8-year biochemical relapse-free rate (BRF) was 92, 91, 89, and 79%. GRADE assessment results indicated that the certainty of the evidence was very low. Meta-regression results did not show a significant relationship based on the variables studied (P<0.05).

**Conclusions:**

Currently available evidence demonstrated that the efficacy and safety of CIRT and PBT for prostate cancer were similar, and they may significantly improve the OS, LCR, and reduce the incidence of GU and GI toxicity compared with photon radiotherapy. However, the quantity and quality of the available evidence are insufficient. More high-quality controlled studies are needed in the future.

## 1 Introduction

Prostate cancer is the most common urologic cancer with the largest increase in the incidence of all cancers (1, 2). It ranks second most frequent cancer and the fifth leading cause of cancer death in men. According to the cancer statistic in 2018, there were almost 1.3 million new cases of prostate cancer and 359,000 associated deaths worldwide in 2018 (3).

Radiation therapy (RT) could be an excellent treatment option for prostate cancer. The outcomes of RT for prostate cancer have improved over the years due to the introduction of new treatment modalities, such as conventional RT, three-dimensional conformal RT, and intensity-modulated RT (IMRT) (4–6). Recently, the use of radiation for treating prostate cancer has increased by approximately 10% compared with previous Japanese studies (7). However, these radiotherapy methods may affect healthy tissues and increase the risk of severe injury to critical organs.

Particle therapy mainly includes carbon-ion RT (CIRT) and proton beam therapy (PBT) and has been used for prostate cancer over the past two decades (8). According to the statistics of the Particle Therapy Co-Operative Group (PTCOG), by end of 2020, more than 290,000 patients have been treated worldwide with particle therapy, close to 250,000 with PBT, and close to 40,000 with CIRT (9). CIRT and PBT offer unique biological and physical advantages over conventional RT with X-rays. Carbon-ion beams have an estimated threefold higher relative biological effectiveness (RBE) than X-rays (10). Regarding the physical aspect, the carbon-ion and proton beam can create a better dose distribution based on the ability of accelerated ions to release a maximum amount of energy at the end of their track, resulting in a Bragg peak (11). These features can permit dose escalation for tumors with less toxicity in normal tissues. Favorable clinical outcomes of CIRT and PBT for prostate cancer have been reported (12–14) but have remained a subject of controversy.

Some studies have reported excellent disease control and favorable toxicity of CIRT and PBT for prostate cancer (2, 15–19). However, most trials recruit a small sample size, and the overall results have remained mixed or inconclusive. Evidence-based research can better support clinical practice (20, 21). A meta-analysis (22) published in 2016 has comprehensively analyzed the efficacy and safety of CIRT for prostate cancer. However, only six studies were included in that article, and the results were not compared with other radiotherapy methods, so it is difficult to systematically evaluate the advantages of CIRT. Moreover, the quality of published trials has not been evaluated, which is an indispensable step before treatment recommendations can confidently be made.

To fill this gap, the systematic review and meta-analysis thus aims to collect and analyze current available scientific evidence on the efficacy and toxicity after CIRT and PBT for prostate cancer, identifying the related studies and characterizing the evidence that will benefit the clinical practice and future high-quality research.

## 2 Material and Methods

Our methods and reporting followed the Preferred Reporting Items for Systematic Reviews and Meta-analyses (PRISMA) (23) and Cochrane handbook (24, 25).

### 2.1 Literature Search

Systematic retrieval of PubMed, EMBASE, Web of Science, Cochrane Library was conducted to collect relevant studies on CIRT for prostate cancer. Research data were restricted from January 2010 to July 2021. We did free-text terms and Mesh searches for the following terms: particle*, heavy ion*, carbon, C-ion, proton*, prostatic neoplasm*, prostate neoplasm*, prostate cancer*, prostatic cancer*, prostate tumor*, prostatic tumor*. The search was restricted to human studies, but no restrictions were placed on language or publication status. We also searched Google Scholar to find gray literature. Additionally, we manually reviewed the reference lists from included studies and relevant systematic reviews to identify other potential studies. The detailed research strategy is shown in **Supplementary Material 1**.

### 2.2 Literature Selection and Criteria

All the retrieved articles were imported into the EndNote X9 software, and the duplicate publications were excluded. Six reviewers (ML, XH, WY, YL, YF, MH, YW), working independently in teams of three, screened all titles and abstracts of retrieved citations, evaluated potential full texts, and determined eligibility. Disagreements were resolved through discussion and consensus or by consulting a third member (XL) of the review team. We included studies in the analysis if they met several criteria:

Types of Study Design: All types of primary studies.

Population: Studies including men (≥18 years of age) diagnosed with prostate cancer (any stage) or mixed cancers were eligible if separate data for men with prostate cancer were available.

Intervention: Treatment group intervention was CIRT or PBT alone or combined with other therapies.

Comparators: Control group intervention was photon radiotherapy including conventional RT, two- or three-dimensional conformal RT, IMRT, and so on.

Outcomes: Overall survival (OS), local control rate (LCR), biochemical relapse-free rate (BRF), gastrointestinal (GI), and genitourinary (GU) toxicity.

If publications were derived from the same population and reported the same associated outcomes, we included only the latest published data or results with the largest number of individuals in our analysis.

We excluded review articles, editorials, comments, and irrelevant topic studies.

### 2.3 Data Abstraction

After pilot testing our data extraction forms, paired reviewers (ML and YL) independently extracted study characteristics and outcomes for each trial. The main contents of data extraction included (1) general information: author, year of publication, country; (2) PICOS characteristics, such as tumor stage, treatment duration, total dose, segmentation times, control intervention, outcomes (OS, LCR, BRF, GI, and GU toxicity), study design; (3) information on relevant items of quality assessment.

### 2.4 Risk of Bias Assessment

We independently assessed the risk of bias of individual studies by two reviewers (ML and LY) using different tools according to different types of study design.

The risk of bias of randomized controlled trials (RCTs) was assessed by Cochrane Handbook v.5.1.0 (26), including seven aspects: Random sequence generation (selection bias), Allocation concealment (selection bias), Blinding of participants and personnel (performance bias), Blinding of outcome assessment (detection bias). Every item was classified as yes (“low risk of bias”), no (“high risk of bias”), or unclear (“moderate risk of bias”). When the risk of bias of all seven items was defined as low risk of bias, the trial was defined as “low risk of bias”; when one or more of the items were classified as high risk, the trial was graded as “high risk of bias.” In other cases, the trial was graded as “Unclear risk.” Any conflict in bias classification was resolved by discussion or, if necessary, through adjudication by a third reviewer (XL or KY).

The risk of bias of cohort studies was evaluated according to criteria developed by the Newcastle Ottawa Scale (NOS) (27). The items included Representativeness of the exposed cohort, Selection of the non-exposed cohort, Ascertainment of exposure, Demonstration that outcome of interest was not present at the start of the study, Comparability of cohorts based on the design or analysis, Assessment of outcome, Whether follow-up was long enough for outcomes to occur, Adequacy of follow-up of cohorts. The risk of bias of case series studies was assessed using a comprehensive quality assessment tool developed by the Institute of Health Economics (IHE) in 2012 (28). This tool includes seven domains and 20 items. The evaluation contents include research purpose, research topic, intervention measures, outcome index measurement, statistical analysis, results and conclusions, conflict of interest, and source of funds. If more than 14 items (70%) are assessed as “yes” in the included studies, the acceptable quality is considered.

If there were any differences in the above evaluation process, they can be resolved through discussion between the two groups of researchers or by consulting a third party.

### 2.5 Certainty of Evidence Assessment

Paired reviewers (ML, XL) independently rated the certainty (quality) of evidence using the Grades of Recommendation, Assessment, Development, and Evaluation (GRADE) system (29) and constructed a summary of the findings table. The GRADE approach was used to assess the quality of a body of evidence based on the extent to which one can be confident that an estimate of effect or association reflects the item being assessed. Assessment of the quality of evidence of observational study considers the risk of bias, inconsistency, indirectness, imprecision, publication bias, large effect, plausible confounding, and dose-response gradient (30).

### 2.6 Statistical Analysis

For the single-arm studies, all outcomes reported incidence rate in a group of patients, R 4.0.2 software (R-4.0.2, 64 bit, The Cochrane Collaboration, Oxford, UK) was used to do a single-arm meta-analysis. This analysis took study effects into account, and the results were calculated by a binary random-effect method (Dersimonian-Laird). Forest plots were used to illustrate the prevalence with a 95% confidence interval. A random-effects model was used for pooling studies because it considers the almost inevitable natural variation inherent between studies. The level of the meta-analysis was set as α = 0.05. Heterogeneity was assessed using the Cochran Q test and I^2^ statistics. In case of heterogeneity among the studies, meta-regression and subgroups analyses were performed. Subgroup analyses were conducted according to different follow-up duration and severity grades of toxicity. The meta-regression analysis mainly included the study design and tumor stage. If the number of variables pooled for an outcome was at least 10, publication bias was assessed through the generation of a funnel plot.

Considering that most of the included studies were single-arm trials without a control group, we cited a published meta-analysis (31) of photon therapy as a reference. We compared results of our meta-analyses of CIRT and PBT with the results of photon therapy from the cited published meta-analyses.

## 3 Results

### 3.1 Literature Search Results and Characteristics

**Figure 1:**
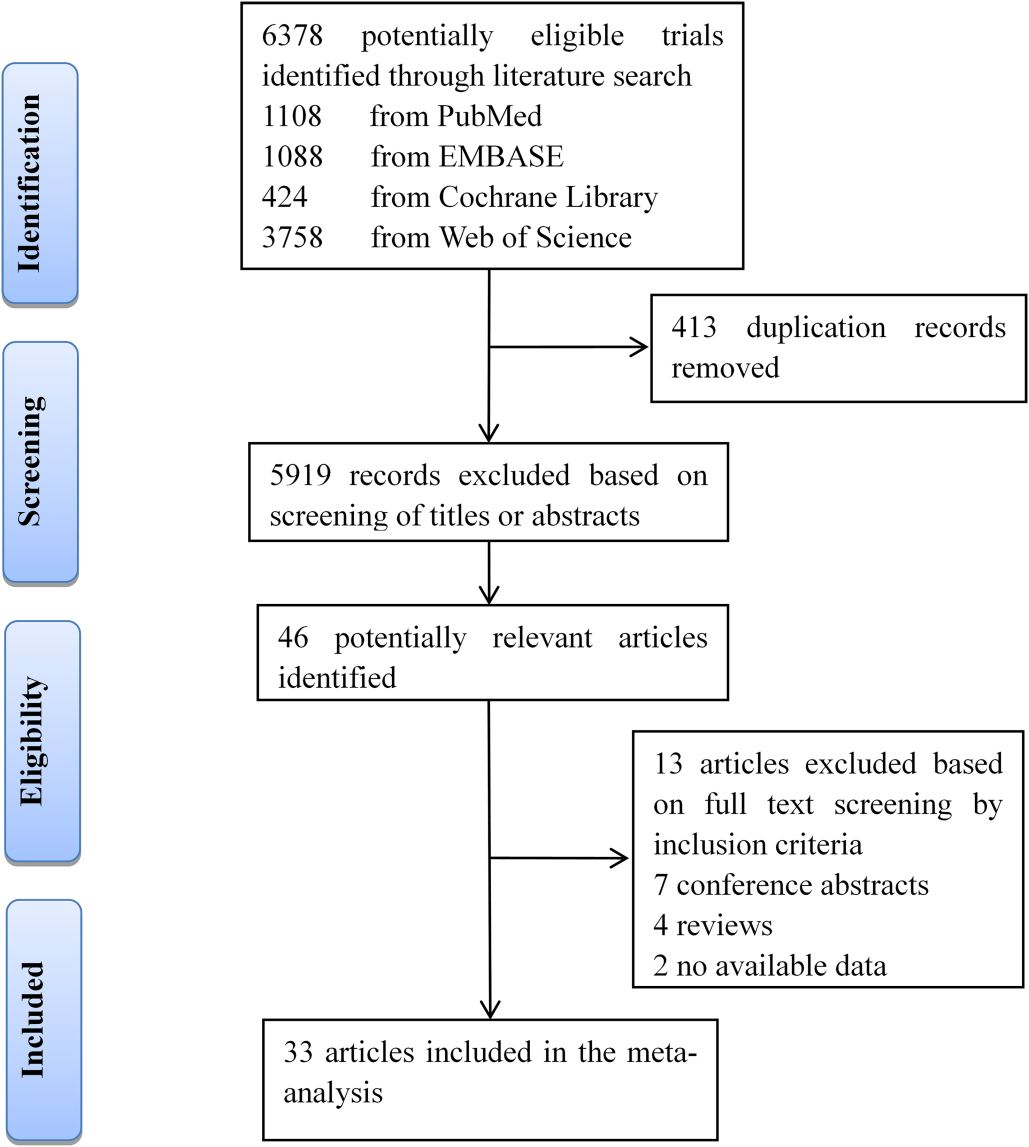
Flow diagram of the literature screening process and results.

Our searches of four databases yielded 6,378 articles. After 413 duplication records were removed, titles and abstracts of these records were screened for inclusion. Full texts of 46 records were read, and 33 studies (13 about CIRT and 20 focused on PBT) met the inclusion criteria (**Figure 1**).

Thirteen (12, 16, 17, 19, 32–40) CIRT for prostate cancer-related studies including one randomized controlled trial (17) and 12 observational studies were included. All of the included studies involved patients from 35 to 2,157 (total 5,336) with a median mean age of 68 years. Median follow-up across all studies was 43 months (range 21–84 months). The included studies were published from 2010 to 2020, including one from China (39), two from Germany (17, 33), and the remaining 10 reports were from Japan. Among the included studies, only one randomized controlled trial (17) compared the safety and efficacy of carbon ion radiotherapy and proton radiation therapy for prostate cancer, one phase I/II clinical trial (33) analyzed the efficacy and safety of CIRT combined with proton radiation therapy for prostate cancer, and the other studies were single-arm trials that only discussed the effectiveness and safety of carbon ion radiotherapy alone for prostate cancer. In terms of irradiation dose, for the trials from Japan, the irradiation dose was usually set at 51.6–66 GyE delivered in 16–20 fractions over 5 weeks. Protocol9402 utilized a total dose of 54–72 GyE across 20 fractions over 5 weeks; Protocol1002 and a new study published in 2020 utilized a total dose of 51.6 GyE across 12 fractions over 3 weeks. For the two trials (17, 33) from Germany, one used 60 GyE across 20 fractions over 5 weeks, and another used 66 GyE across 20 fractions over 3.5 weeks. A study from China (39) set irradiation dose at 59.2–66 GyE delivered in 24 fractions over 5 weeks.

Twenty PBT-related studies (13, 14, 18, 41–57) involved 48,765 patients with a median mean age of 66 years old. Median follow-up across all studies was 43.4 months (range 6–85.2 months). The included studies were published from 2010 to 2020. Most of the studies were from the USA (n=14), five from Japan (14, 18, 47, 52, 53), and one from Korea (48). For the trials from the USA, most of the studies set irradiation dose at 70–82 GyE delivered in 5–44 fractions. For the five trials from Japan, the irradiation dose was usually set at 63–80 GyE delivered in 20–39 fractions. For one trial from Korea, they set irradiation dose at 35–60 GyE delivered in 5–20 fractions. The basic characteristics of the included studies are shown in **Table 1**.

**Table 1:** Basic characteristics of included studies.

Trial	Study ID	Country	Study design	Intervention	Tumor stage	Duration	Sample Size	Mean/Median Age (year)	Radiotherapy details			median follow-up (months)	Outcomes
									Total dose (GyE)	Fraction	Duration (week)		
Protocol9402	Shimazaki, 2010 (32)	Japan	Retrospective phase I/II trial	CIRT	T2b-T3N0M0	1995-2000	35	69	54-72	20	5	47	OS,BRF,LCR,GI,GU
Protocol9703	Shimazaki, 2010 (32)	Japan	retrospective phase I/II trial	CIRT	T1b-T2N0M0	1995-2000	61	69	60-66	20	5	84	OS,BRF,LCR,GI,GU
Protocol9904(a)	Shimazaki, 2010 (32)	Japan	retrospective phase I/II trial	CIRT	T1b-T2a	2000-2003	175	70	60-66	20	5	43	OS,BRF,LCR,GI,GU
NR	Nikoghosyan, 2011 (33)	Germany	prospective phase I/II trial	CIRT + IMRT	T1-3	2006-2008	14	68	60	20	5	28	GI,GU
Protocol9904(b)	Hitoshi, 2012 (34)	Japan	retrospective phase II trial	CIRT	T1b-T3bN0M0	2003-2005	120	67.6	66	20	5	21	OS,BRF,LCR
Protocol9904-2	Hitoshi, 2012 (34)	Japan	retrospective phase II trial	CIRT	T1b-T3bN0M0	2005-2007	171	67.6	63	20	5	29	OS,BRF,LCR
Protocol9904-3	Hitoshi, 2012 (34)	Japan	retrospective phase II trial	CIRT	T1b-T3bN0M0	2007-	520	67.6	57.6	20	5	43	OS,BRF,LCR
Protocol9904	Okada, 2012 (35)	Japan	retrospective phase II trial	CIRT	T1b-T3	2000-2009	664	68.2	63-66	20	5	24	OS,BRF,GI,GU
Protocol9904	Katoh, 2014 (36)	Japan	prospective study	CIRT	T1c-T3bN0M0	2000-2004	213	69.4	66	20	5	36	OS,BRF,GI,GU
Protocol1002	Nomiya, 2014 (37)	Japan	prospective phase I/II trial	CIRT	T1b-T3bN0M0	2010-	46	66	51.6	12	3	32.3	OS,GI,GU
ProtocolGUNMA0702 Protocol/ GUNMA0702EX	Hitoshi, 2015 (38)	Japan	prospective phase II trial	CIRT	T1c-T4	2010-2011	76	66	57.6	16	5	51	OS,BRF,GI,GU
NR	Habl, 2016 (17)	Germany	RCT	PBT vs CIRT	T1c-T3bN0M0	2012-2013	46	68	66	20	3.5	22.3	GI,GU,QOL
J-CROS 1501PR	Nomiya, 2016 (12)	Japan	prospective phase II trial	CIRT	T1c-T3bN0M0	2003-2014	2157	67	51.6-66	20	5	43	OS,BRF,LCR,GI,GU
Protocol9904	Maruyama, 2017 (19)	Japan	prospective phase II trial	CIRT	T1-3	2000-2007	417	69	63-66	20	5	60	QOL
NR	Zhang, 2019 (2)	China	retrospective observational study	C-RT	T1-T4	2015-2018	64	70.5	59.2-66	24	5	24	GI,GU
GUNMA0702	Kawamura, 2020 (40)	Japan	prospective observational study	CIRT	T1–T3N0M0	2010-2013	304	66	57.6	16	4	60	BRF,LCR,OS,GI,GU
NR	Takakusagi, 2020 (16)	Japan	retrospective observational study	CIRT	T1bN0M0-T3bN0M0	2015- 2017	253	70	51.6	12	3	51.6	BRF,GI,GU
NR	Nihei, 2011 (53)	Japan	Prospective phaseI/II	PBT	T1c- T3a	2004-2007	151	67	74	37	NR	43.4	GI,GU
NR	Yu,2012 (57)	USA	Retrospective observational study	PBT VS IMRT	NR	2008-2009	27647	66-94	NR	NR	NR	12	GI,GU
protocols PR-01 (UFJ-2005154); PR-02 (UFJ-2006-63); PR-03 (UFJ-2006-94)	Mendenhall, 2013 (50)	USA	Prospective observational study	PBT	NR	2006-2007	211	68	78-82	39	NR	62.4	OS,GI,GU
protocol PR-01/02	Henderson, 2013 (45)	USA	Prospective observational study	PBT	NR	2006-2007	171	66	74	39	NR	43.4	GU
NR	Kim, 2013 (48)	Korea	Prospective phaseII	PBT	T1-T3N0M0	NR	82	68	35-60	5-20	5	42	GI,GU
NR	Fang, 2014 (43)	USA	Case-Matched Study	PBT vs IMRT	NR	PBT:2010-2012 IMRT:2009-2012	394	³40	79.2	44	NR	47	GI,GU
protocol (OTP)	Bryant, 2016 (41)	USA	Prospective observational study	PBT	T1-T3	2006-2010	1327	66	³75	26	NR	63.6	OS,GI,GU
NR	Vargas, 2016 (56)	USA	Prospective observational study	PBT	T1- T2	NR	49	65	38	5	NR	18	GI,GU
protocol, PR04	Henderson, 2017 (44)	USA	Prospective observational study	PBT	T1–T2b	2008-2011	215	65	70-72.5	28-29	9.5	62.4	OS,GI,GU
NR	Iwata, 2017 (47)	Japan	Retrospective observational study	PBT	T1-T4	2008-2011	1291	68	70-80; 63-66	35-40;21-22	NR	69	OS, BRF,GI,GU
NR	Nakajima, 2018 (52)	Japan	Prospective phaseI/II	HFPT vs CFPT	T1–T3N0M0	2013-2016	526	69.5	63-78	CFPT20-37 HFPT:21-39	CFPT:7.8;HFPT:4.2	³6	GI,GU
NR	Arimura, 2018 (18)	Japan	Prospective observational study	PBT	T2a-T3b	2011-2014	204	65	70;74;78	28;37;39	NR	52	OS,GI,GU
Medicare claims	Pan, 2018 (54)	USA	Retrospective observational study	PBT vs SBRT vs IMRT	NR	2008-2015	12128	≤65	NR	IMRT: 42; proton radiation: 39 ; SBRT:5	NR	23	GI,GU
NR	Ho, 2018 (46)	USA	Prospective observational study	PBT	T1c -T3a	2006-2010	254	56	70-82	2-2.5 Gy/fraction	NR	85.2	OS
NR	Chuong, 2018 (42)	USA	Prospective observational study	PBT	T1-T3	2010-2016	85	69	70–80.2	44	NR	14.5	GI,GU
NR	Santos, 2019 (55)	USA	Retrospective observational study	PBT VS IMRT	NR	2009-2017	307	59.7	66.0-70.2	1.8-2.0-Gy/fraction	NR	46.1	GU,GI
NR	Slater, 2019 (13)	USA	Prospective phaseI/II	PBT	T1-T2a	2009-2017	167	65	60	20	4	42	GU,GI
PCG 001-09	Mishra, 2019 (51)	USA	Prospective observational study	PBT	T1-T2c	2009-2017	1343	66.6;65.2	79.3; 79.4	NR	NR	16.5; 27.2	GI, GU,
NR	Lee, 2019 (49)	USA	Retrospective observational study	PBT	T1-T4	2013-2016	192	68	79.2	44	NR	20	GI
NR	Takagi, 2020 (14)	Japan	Retrospective observational study	PBT	NR	2003-2014	2021	68	74	37	NR	84	BRF, OS, GI, GU

CIRT, carbon ion radiotherapy; PBT, proton beam therapy; OS, Overall survival; LCR, Local control rate; GI, Gastrointestinal toxicity; GU, Urinary tract toxicity; QOL, Quality of life; IMRT, intensity modulated radiation therapy; HFPT, Hypofractionated proton therapy; CFPT, conventional fractionated proton therapy; SBRT, stereotactic body radiotherapy. NR, not reported.

### 3.2 Risk of Bias and Certainty of the Evidence Assessment Results

**Figure 2:**
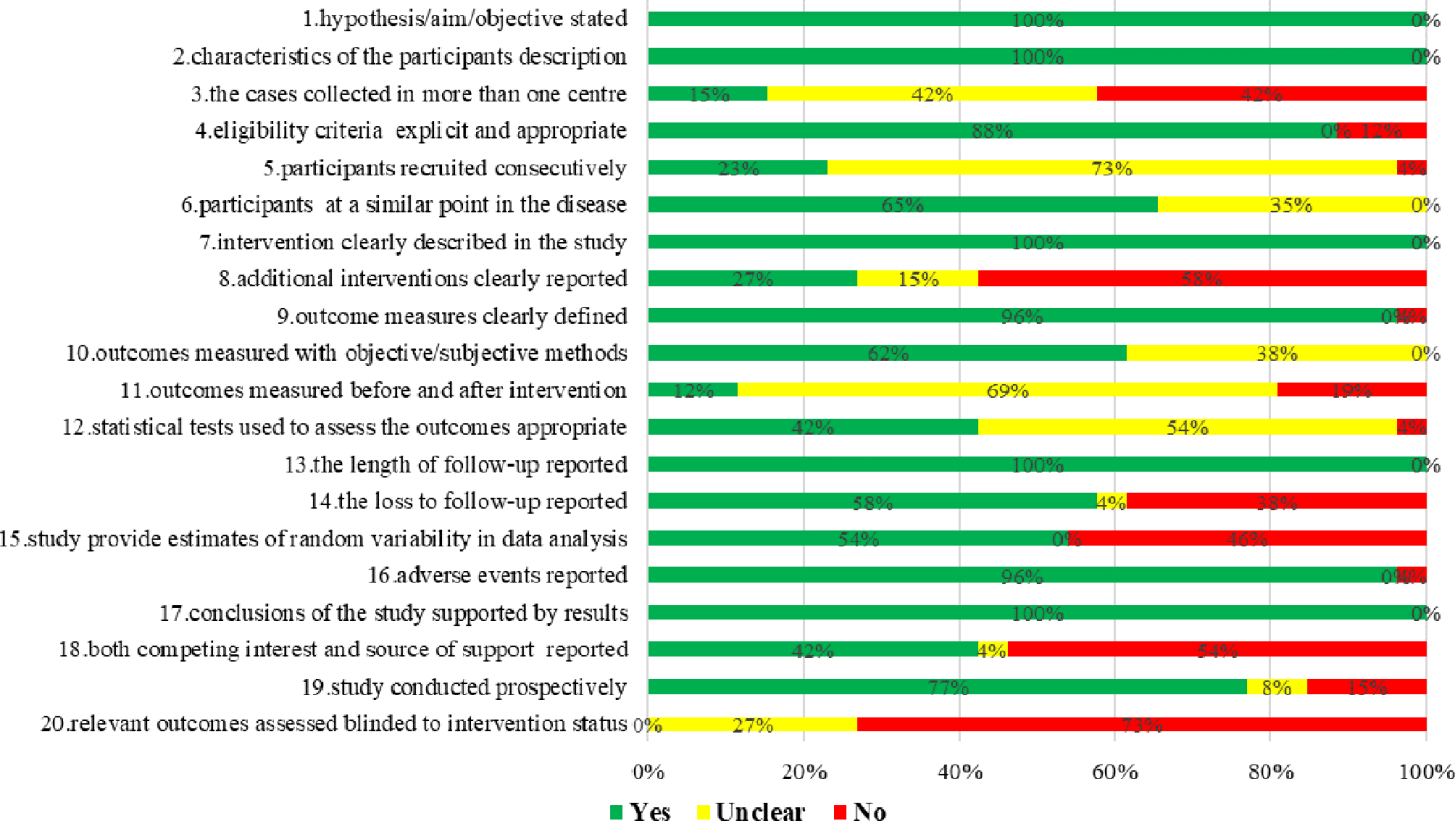
Risk of bias assessment of case series studies of proton and carbon ion radiotherapy for prostate cancer.

IHE quality assessment results showed that the overall quality of included case series studies was low, mainly due to none of the studies reported blinding of outcome assessors; only three studies (19, 33, 46) reported measured outcomes before and after the intervention; four studies (12, 42, 47, 53) reported collecting cases in multiple centers; and six studies (16, 18, 38, 39, 44, 49) reported that participants recruited consecutively (**Figure 2**).

NOS quality assessment results indicated that the risk of bias of the included six cohort studies was low, with quality score 7–9 (**Supplementary Material 2**).

The results of the ROB evaluation showed that the risk of bias in a randomized controlled trial (17) was unclear. In this RCT, “Random sequence generation,” “Allocation concealment,” “Blinding of participants and personnel,” and “Blinding of outcome assessment” was not reported. “Incomplete outcome data,” “Selective reporting,” and “Other bias” were judged as low-risk bias.

GRADE assessment results showed the certainty of the evidence was low or very low, mainly because of the risk of bias, high heterogeneity between studies (inconsistency), and wide confidence intervals (imprecision) (**Table 2**).

**Table 2:** Summary of findings of carbon ion radiotherapy and proton beam therapy for prostate cancer patients.

Outcomes	Carbon ion radiotherapy		Proton Beam Therapy	
	№ of participants (studies)	Certainty of the evidence (GRADE)	№ of participants (studies)	Certainty of the evidence (GRADE)
OS follow up: range 36 months to 120 months	2307 (8 observational studies)	⊕⃝⃝⃝ VERY LOW	11417 (7 observational studies)	⊕⃝⃝⃝ VERY LOW
LCR follow up: range 36 to 60 months	1004 (6 observational studies)	⊕⊕⃝⃝ LOW	–	–
BRF follow up: range 36 months to 96 months	2211 (8 observational studies)	⊕⃝⃝⃝ VERY LOW	–	–
acute gastrointestinal toxicity (AGI) follow up: range 6 months to 96 months	7753 (8 observational studies)	⊕⃝⃝⃝ VERY LOW	4057 (8 observational studies)	⊕⃝⃝⃝ VERY LOW
(LGI) follow up: range 6 months to 96 months	11304 (12 observational studies)	⊕⃝⃝⃝ VERY LOW	10856 (12 observational studies)	⊕⃝⃝⃝ VERY LOW
AGU follow up: range 6 months to 96 months	10038 (9 observational studies)	⊕⃝⃝⃝ VERY LOW	6164 (12 observational studies)	⊕⃝⃝⃝ VERY LOW
LGU follow up: range 6 months to 96 months	12384 (12 observational studies)	⊕⃝⃝⃝ VERY LOW	11575 (15 observational studies)	⊕⃝⃝⃝ VERY LOW

OS, overall survival; LCR, local control rate; BRF, biochemical relapse-free rate; AGI, acute gastrointestinal toxicity; LGI: late gastrointestinal toxicity; AGU, acute genitourinary toxicity; LGU, late genitourinary toxicity.

**GRADE Working Group grades of evidence**

**High certainty:** We are very confident that the true effect lies close to that of the estimate of the effect.

**Moderate certainty:** We are moderately confident in the effect estimate: The true effect is likely to be close to the estimate of the effect, but there is a possibility that it is substantially different.

**Low certainty:** Our confidence in the effect estimate is limited: The true effect may be substantially different from the estimate of the effect.

**Very low certainty:** We have very little confidence in the effect estimate: The true effect is likely to be substantially different from the estimate of effect.

### 3.3 Meta-Analysis

#### 3.3.1 Overall Survival

**Figure 3:**
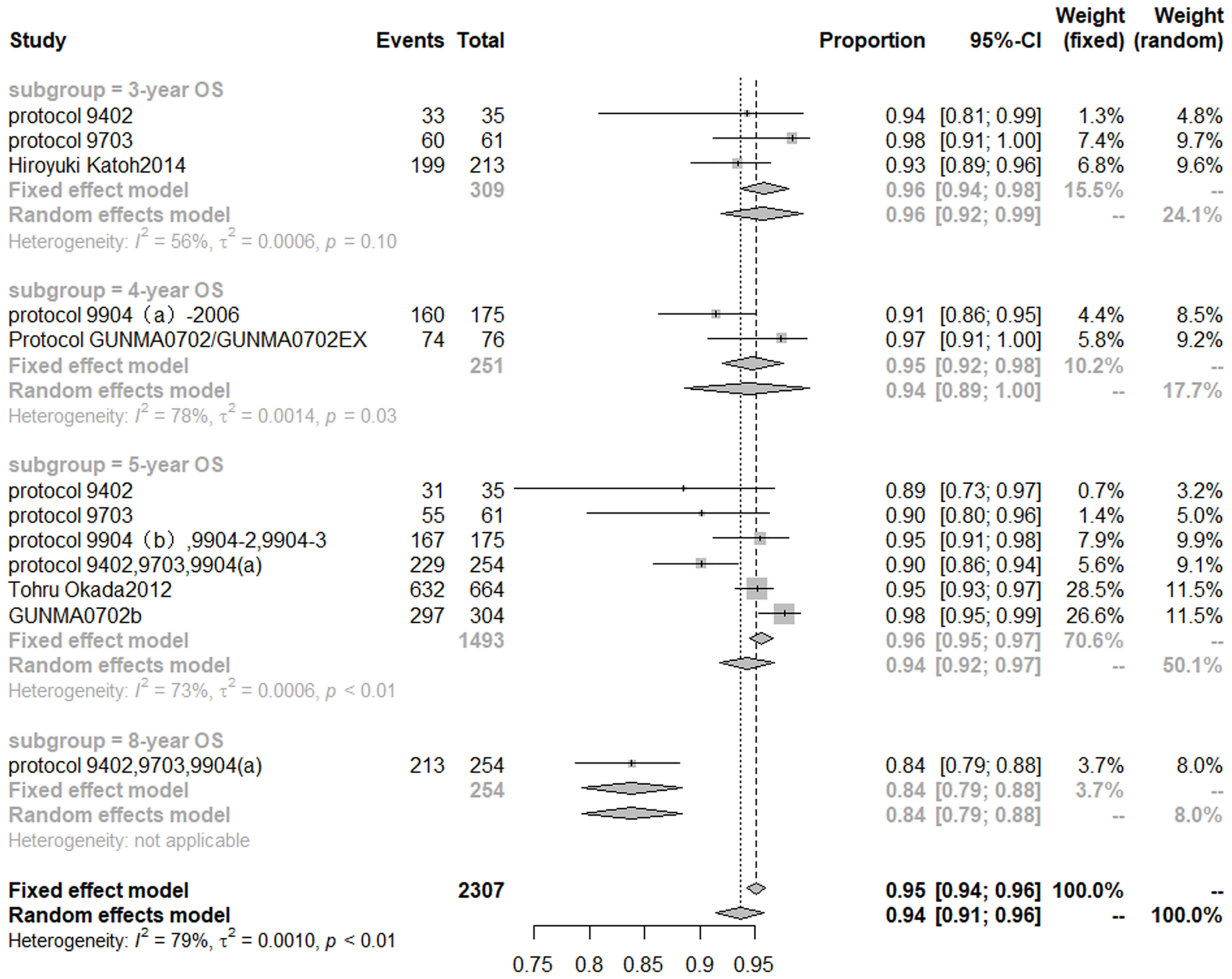
Overall survival rate of carbon ion radiotherapy for prostate cancer.

Based on the random effect model after inclusion of eight studies (32, 34–36, 40, 58–60), the 3-, 4-, 5-, 8-year OS of CIRT for prostate cancer was 96% (95% CI, 92–99%), 94% (95% CI, 89–100%), 94% (95% CI, 92–97%), 84% (95% CI, 79–88%), respectively. Cochran Q statistics show 56, 78, and 73% heterogeneity, respectively, among studies in 3-, 4-, 5-year OS (**Figure 3**).

**Figure 4:**
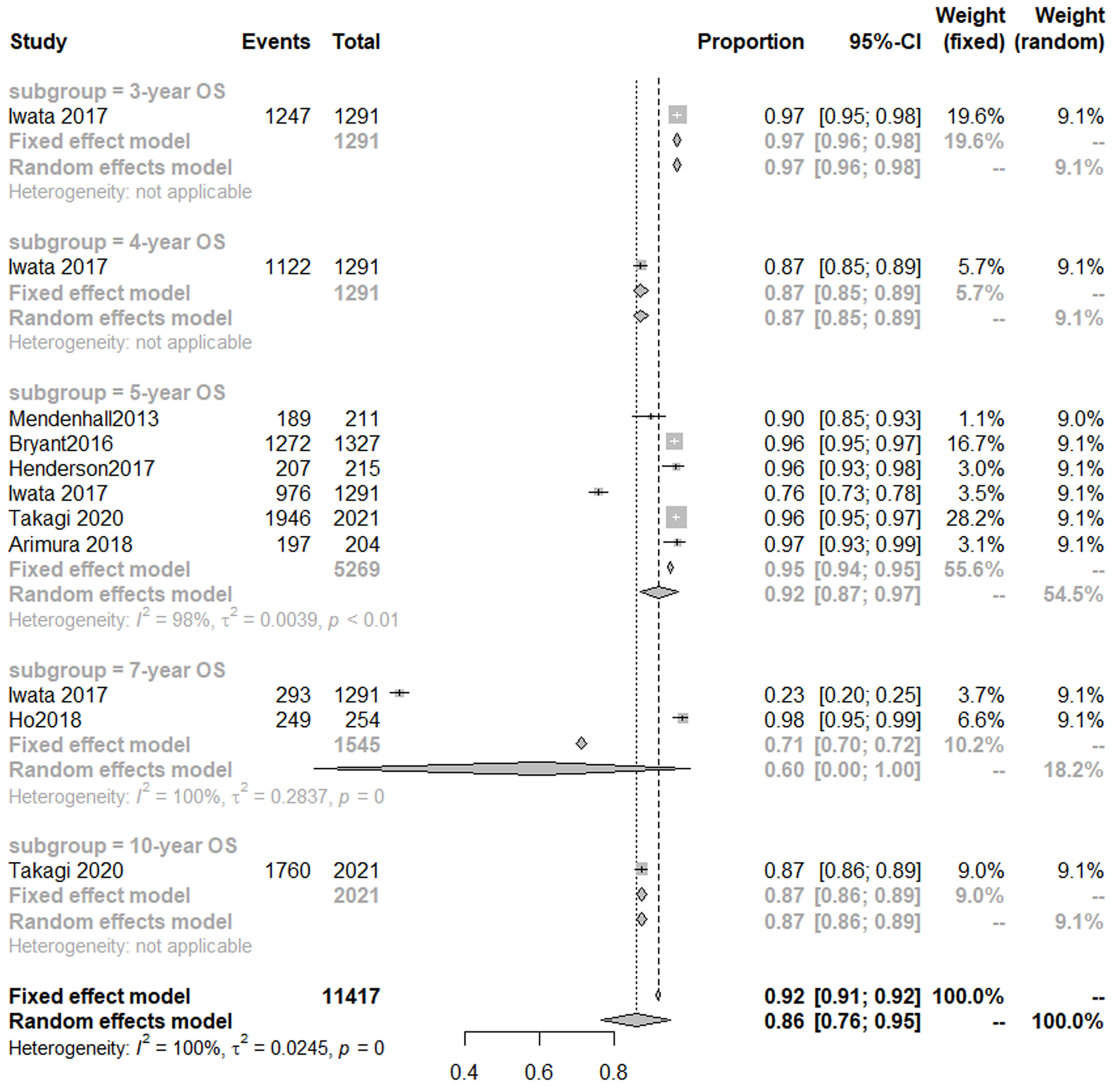
Overall survival rate of protons radiotherapy for prostate cancer.

Seven studies (14, 18, 41, 44, 46, 47, 50) analyzed OS of PBT for prostate cancer. A random-effect meta-analysis indicated that the 3-, 4-, 5-year OS was 97% (95% CI, 96–98%), 87% (85–89%), 92% (95% CI, 87–97%), respectively (**Figure 4**).

The results of a meta-analysis (31) published in 2017 showed that the 5-year OS of patients with prostate cancer treated with conventional photon radiotherapy was 72% (1,854/2,583), and hypofractionated photon radiotherapy was 72.8% (1,897/2,605). Noth of them was lower than that of patients treated with CIRT and PBT.

#### 3.3.2 Local Control Rate

Six studies (five protocols) (32, 34, 40, 58–60) evaluated LCR of CIRT for prostate cancer. Random-effects model single-arm meta-analyses showed that the 3-, 4-, 5-year LCR of CIRT for prostate cancer was 98% (95% CI, 95–100%), 97% (95% CI, 95–100%), and 99% (95% CI, 98–99%), respectively. I^2^ was 0% among studies of 3-, 4-, 5-year LCR (**Supplementary Figure 1** and **Table 3**).

**Table 3:** Meta-analysis results.

Outcomes	Subgroups	Intervention	No of studies	No of patients	Proportion (%)	95% CI	I^2^(%)
OS	3-year	Carbon	3	309	96	92-99	56
		Proton	1	1291	97	96-98	/
	4-year	Carbon	2	251	94	89-100	78
		Proton	1	1291	87	85-89	/
	5-year	Carbon	6	1493	94	92-97	73
		Proton	6	5269	92	87-97	98
		photon (C-RT)	5	2583	72	/	0
		photon (HRT)	5	2605	73	/	0
	8-year OS	Carbon	1	254	84	79-88	/
AGI	Grade 0	Carbon	7	3044	95	92-98	93
		Proton	2	233	88	84-92	0
	Grade 1	Carbon	6	887	11	5-17	95
		Proton	5	1661	16	12-20	72
	Grade 2 or higher	Carbon	7	3044	1	0-3	95
		Proton	8	2163	1	0-2	66
		photon (C-RT)	6	2258	14	/	53
		photon (HRT)	6	2271	19	/	53
LGI	Grade 0	Carbon	7	1986	90	87-93	75
		Proton	2	233	56	16-96	98
	Grade 1	Carbon	8	3914	19	0-58	100
		Proton	6	3682	18	10-26	97
	Grade 2 or higher	Carbon	9	4218	2	1-3	78
		Proton	11	6941	4	2-5	92
		photon (C-RT)	6	2499	8	/	69
		photon (HRT)	6	2550	10	/	69
AGU	Grade 0	Carbon	7	3044	56	30-81	99
		Proton	2	233	29	23-34	0
	Grade 1	Carbon	7	951	41	25-57	97
		Proton	6	1755	68	62-73	73
	Grade 2 or higher	Carbon	8	3108	5	2-8	88
		Proton	12	4176	13	9-17	97
		photon (C-RT)	6	2258	28	/	62
		photon (HRT)	6	2271	29	/	62
LGU	Grade 0	Carbon	7	3661	66	44-88	100
		Proton	2	233	76	57-94	90
	Grade 1	Carbon	7	1796	29	15-44	98
		Proton	6	3682	20	4-35	99
	Grade 2 or higher	Carbon	9	4029	4	2-7	93
		Proton	15	7660	5	4-7	93
		photon (C-RT)	6	2499	13	/	17
		photon (HRT)	6	2550	14	/	17
LCR	3-year LCR	Carbon	2	96	98	95-100	0
	4-year LCR	Carbon	1	175	97	95-100	/
	5-year LCR	Carbon	3	733	99	98-99	0
BRF	3-year BRF	Carbon	1	213	92	88-95	/
	4-year BRF	Carbon	2	251	91	83-99	79
	5-year BRF	Carbon	6	1493	89	86-92	68
	8-year BRF	Carbon	1	254	79	74-84	/

OS, overall survival; LCR, local control rate; BRF, biochemical relapse-free rate; AGI, acute gastrointestinal toxicity; LGI, late gastrointestinal toxicity; AGU, acute genitourinary toxicity; LGU, late genitourinary toxicity; C-RT, conventional radiotherap; HRT, hypofractionated radiotherapy.

#### 3.3.3 Biochemical Relapse-Free Rate

Eight studies (32, 34–36, 40, 58–60) reported BRF of CIRT for prostate cancer. Random effects model single-arm meta-analyses showed that the 3-, 4-, 5-, 8-year BRF of CIRT for prostate cancer was 92% (95% CI, 88–95%), 91% (95% CI, 83–99%), and 89% (95% CI, 86–92%), 79% (95% CI, 74–84%), respectively. I^2^ was 79 and 68% among studies of 4-, 5-year BRF (**Supplementary Figure 2** and **Table 3**).

#### 3.3.4 Gastrointestinal Toxicity

Eight studies (12, 16, 17, 32, 36–38, 59) reported acute gastrointestinal toxicity (AGI), and nine studies (12, 16, 17, 32, 34–38, 40, 58, 59) provided detailed data on late gastrointestinal toxicity (LGI). The results of a randomized controlled trial (17) showed that the incidence of grade 2 AGI toxicity of proton and heavy-ion radiotherapy was 8.7 and 2.2%, respectively. Random effects model single-arm meta-analyses showed that the grade 2 or more serious AGI and LGI of CIRT for prostate cancer was 1% (95% CI, 0–3%) and 2% (95% CI, 1–3%), and I^2^ was 95 and 78%, respectively (**Supplementary Figures 3**, **4** and **Table 3**).

Eight studies (13, 18, 42, 48, 50, 51, 53, 56) provided sufficient data about AGI of PBT for prostate cancer, and 11 studies (14, 18, 41, 42, 44, 47, 48, 50, 51, 53, 56) reported LGI of PBT for prostate cancer. A random-effects model single-arm meta-analyses showed that the Grade 2 or higher AGI and LGI of PBT for prostate cancer was 1% (95% CI, 0–2%) and 4% (95% CI, 2–5%) (**Supplementary Figures 5**, **6** and **Table 3**).

A meta-analysis (31) showed that the incidence of grade 2–4 AGI toxicity of conventional photon radiotherapy was 14% (314/2,258) and the LGI toxicity was 8% (211/2,499). The incidence of grade 2–4 AGI toxicity of hypofractionated photon radiotherapy was 19% (433/2,271), and the LGI toxicity was 10% (243/2,551). It can be seen that the incidence of GI toxicity of CIRT and PBT was lower than that of conventional photon radiotherapy and hypofractionated photon radiotherapy.

#### 3.3.5 Genitourinary Toxicity

An included RCT (17) reported no patient developed grade 3/4 acute genitourinary toxicity (AGU) in the proton group and CIRT group. Random effects model single-arm meta-analyses of 8 studies (12, 16, 17, 32, 34–38, 40, 58, 59) showed that the grade 2 or more serious AGU of CIRT for prostate cancer was 5% (95% CI, 2%-8%), I^2^ was 97%. Random effects model single-arm meta-analyses of nine studies (12, 16, 17, 32, 34–38, 40, 58, 59) showed grade 2 or more serious late genitourinary toxicity (LGU) of CIRT for prostate cancer was 4% (95% CI, 2–7%); I^2^ was 93% (**Supplementary Figures 7**, **8** and **Table 3**).

Twelve studies (13, 18, 41–43, 45, 48, 50, 51, 53, 55, 56) reported AGU of PBT for prostate cancer, and 15 studies (13, 14, 18, 41–45, 47, 48, 50, 51, 53, 55, 56) focused on LGU of PBT for prostate cancer. The random effects model single-arm meta-analyses showed that the grade 2 or higher AGU and LGU of PBT was 13% (95% CI, 9–17%) and 5% (95% CI, 4–7%) (**Supplementary Figures 9**, **10** and **Table 3**).

A meta-analysis (31) showed that the incidence of grade 2–4 AGU toxicity of conventional photon radiotherapy was 28% (627/2,258), and the LGU toxicity was 13% (328/2,499). The incidence of grade 2–4 AGU toxicity of hypofractionated photon radiotherapy was 29% (666/2,271), and the LGU toxicity was 14% (367/2,550). The incidence of GU toxicity of CIRT and PBT was lower than that of conventional photon radiotherapy and hypofractionated photon radiotherapy.

### 3.4 Meta-Regression and Publication Bias

Meta-regression results did not show a significant relationship based on the variables studied. For all outcomes, based on the study design, no significant relationship was obtained. All P values were greater than 0.05. Also, the results of meta-regression between the tumor stage and the outcomes of CIRT and PBT for prostate cancer did not manifest a significant relationship. All P values were greater than 0.05.

Ten or more studies reported LGI, AGU, and LGU of PBT for prostate cancer, so we did publication bias analyses for the three outcomes. The two sides of the funnel plots were not stacked, indicating the possibility of publication bias (see **Supplementary Figure 11**).

## 4 Discussion

This is the first English language meta‐analysis of CIRT and PBT for prostate cancer patients reported to date. We identified 33 published studies including a total of 54,101 patients from the USA, Japan, Germany, China, and Korea. Based on the available evidence, we found that compared with photon therapy, CIRT and PBT for prostate cancer had higher OS and LCR, with both over 90%, and lower incidence of grade 2 or greater GI and GU toxicity, ranging from 1 to 13%. In particular, the advantages of carbon-ion radiotherapy are more prominent, 5-year OS was 94%, and the incidence of grade 2 or greater GI and GU toxicity ranged from 1 to 5%. The quality of the included studies was found to be either low or moderate quality, and the certainty of the evidence was very low.

The promising aspect of CIRT and PBT for cancer therapy lies in the superior biological dose distribution that makes the carbon ion and proton beam the best-balanced particle beam available (60). According to the meta-analysis published in 2016 (22), the 5-year OS rate of patients with prostate cancer treated by CIRT was 91.8%. The results of our meta-analysis showed that the 5-year OS rates of prostate cancer patients treated with CIRT and PBT were 94 and 92%, respectively. The consistency of the two studies confirmed the significant advantage of CIRT and PBT for prostate cancer. A meta-analysis (31) published in 2017 indicated that the 5-year OS rate of prostate cancer patients treated with conventional photon radiotherapy and moderate hypofractionated photon radiotherapy were 72% (1,854/2,583) and 73% (1,897/2,605). Both of them were lower than that of patients treated with CIRT and PBT; the difference between the groups was statistically significant.

GI and GU toxicity is often a problem with RT for prostate cancer. CIRT and PBT can more strongly reduce the rectal dose than other radiotherapy based on its sharp dose distribution to the target. In this study, we found the incidence of grade 2 or greater GI and GU toxicity of CIRT for prostate cancer was ranged from 1–5% and PBT with 1–13%. According to the previous studies, in patients with prostate cancer, grade 2 or greater LGI toxicity was observed 14–24% treated with high-dose 3DCRT (61, 62), and 5–15% using IMRT to spare the rectal dose (4, 63, 64). A meta-analysis (31) showed that the incidence of grade 2–4 GI and GU toxicity in conventional photon radiotherapy was ranged from 8 to 28% and in hypofractionated photon radiotherapy was 10–29%. It can be seen that the incidence of grade 2 or greater GI and GU toxicity of photon radiotherapy is higher than that of CIRT and PBT.

In the aspect of quality of included studies, the published clinical trials of CIRT for prostate cancer were low, and there was still room for improvement in the future. The quality evaluation results of the included studies showed that the following aspects should be paid attention to in future clinical trials: firstly, the recruitment of patients should come from multiple centers; secondly, whether there were additional interventions should be reported in detail; thirdly, the conflict of interest and funding sources should be reported clearly (65–67); lastly, the importance of the implementation of the blind method for patients needs to be emphasized in trials (68, 69). Improvements in the above aspects can greatly reduce the risk of bias in the research results (70). In addition, most of the studies we included were from Japan and the USA, with a few from Germany, China, and Korea, and the publication bias analysis results showed that there might exist publication bias, so the results should be interpreted with caution.

Strengths of this review include our use of explicit eligibility criteria; conducted a comprehensive literature search developed with an experienced librarian; performed a duplicate assessment of study eligibility, risk of bias, and data extraction; summarized the data using a transparent statistical analysis. This transparent and detailed analysis of existing evidence of efficacy and safety of CIRT and PBT for prostate cancer has reference significance for future research and clinical practice. But there are still some limitations: Firstly, most of the included studies in the meta-analysis were single-arm Phase I/II clinical trials and there were few controlled studies on CIRT and PBT for prostate cancer; therefore, we cannot compare the advantages and disadvantages of CIRT and PBT with other treatment methods based on a balanced baseline. Secondly, clinical studies on prostate cancer treatment using CIRT and PBT are limited, and most of the included case series studies had low quality, and the certainty of the evidence was also very low, so the results should be interpreted with caution.

## 5 Conclusions

This meta-analysis found that compared with photon radiotherapy, CIRT and PBT for prostate cancer may improve the overall survival rate and local tumor control rate, and reduce the toxicity of the urinary and gastrointestinal tract, so they have a good application prospect. In the future, more high-quality controlled studies are needed to further analyze the advantages of carbon and proton radiotherapy over other treatments.

## Data Availability Statement

The original contributions presented in the study are included in the article/**Supplementary Material**. Further inquiries can be directed to the corresponding authors.

## Author Contributions

Conception and design: KY, XW, QZ, and XL. Search and collection of data: ML, WY, YL, YF, YW, and MH. Data analysis and interpretation: ML, LY, XL. Manuscript writing: ML and XL. All authors contributed to the article and approved the submitted version.

## Funding

Supported by the National Social Science Fund of China (no. 19ZDA142) and Key Laboratory of Evidence-Based Medicine and Knowledge Translation Foundation of Gansu Province (no. GSEBMKT-2020KF01).

## Conflict of Interest

The authors declare that the research was conducted in the absence of any commercial or financial relationships that could be construed as a potential conflict of interest.

## Publisher’s Note

All claims expressed in this article are solely those of the authors and do not necessarily represent those of their affiliated organizations, or those of the publisher, the editors and the reviewers. Any product that may be evaluated in this article, or claim that may be made by its manufacturer, is not guaranteed or endorsed by the publisher.

## References

[1] Brunckhorst O Hashemi S Martin A George G Van Hemelrijck M Dasgupta P . Depression, Anxiety, and Suicidality in Patients With Prostate Cancer: A Systematic Review and Meta-Analysis of Observational Studies. Prostate Cancer Prostatic Dis (2020) 24(2):281–9. doi: 10.1038/s41391-020-00286-0 32978524

[2] Zhang YF Li P Yu Q Wu S Chen X Zhang Q . Preliminary Exploration of Clinical Factors Affecting Acute Toxicity and Quality of Life After Carbon Ion Therapy for Prostate Cancer. Radiat Oncol (2019) 14(1):94. doi: 10.1186/s13014-019-1303-3 31164172 PMC6549341

[3] Siegel RL Miller KD Jemal A . Cancer Statistics, 2018. CA Cancer J Clin (2018) 68(1):7–30. doi: 10.3322/caac.21442 29313949

[4] Michalski JM Yan Y Watkins-Bruner D Bosch WR Winter K Galvin JM . Preliminary Toxicity Analysis of 3-Dimensional Conformal Radiation Therapy Versus Intensity Modulated Radiation Therapy on the High-Dose Arm of the Radiation Therapy Oncology Group 0126 Prostate Cancer Trial. Int J Radiat Oncol Biol Phys (2013) 87(5):932–8. doi: 10.1016/j.ijrobp.2013.07.041 PMC3840044 24113055

[5] Wilcox S Aherne NJ Benjamin LC Wu B Thomaz DCS Mclachlan CS . Long-Term Outcomes From Dose-Escalated Image-Guided Intensity-Modulated Radiotherapy With Androgen Deprivation: Encouraging Results for Intermediate- and High-Risk Prostate Cancer. Onco Targets Ther (2014) 30(7):1519–23. doi: 10.2147/OTT.S65238 PMC4155897 25210465

[6] Zelefsky MJ Cowen D Fuks Z Shike M Burman C Jackson A . Long Term Tolerance of High Dose Three-Dimensional Conformal Radiotherapy in Patients With Localized Prostate Carcinoma. Cancer (1999) 85(11):2460–8. doi: 10.1002/(sici)1097-0142(19990601)85:11<2460::aid-cncr23>3.0.co;2-n 10357419

[7] Mizuki O Shiro H Taiji T Mototsugu O Osamu O Tadaichi K . Recent Trends in the Initial Therapy for Newly Diagnosed Prostate Cancer in Japan. Jpn J Clin Oncol (2014) 44(10):969–81. doi: 10.1093/jjco/hyu104 25098707

[8] Akakura K Tsujii H Morita S Tsuji H Yagishita T Isaka S . Phase I/II Clinical Trials of Carbon Ion Therapy for Prostate Cancer. Prostate (2010) 58(3):252–8. doi: 10.1002/pros.10328 14743464

[9] Particle Therapy Co-Operative Group . Statistics About Patients Treated With Particles. (2021). Available at: https://www.ptcog.ch/index.php (Accessed July 12, 2021).

[10] Kanai T Endo M Minohara S Miyahara N Koyama-ito H Tomura H . Biophysical Characteristics of HIMAC Clinical Irradiation System for Heavy-Ion Radiation Therapy. Int J Radiat Oncol Biol Phys (1999) 44(1):201–10. doi: 10.1016/s0360-3016(98)00544-6 10219815

[11] Schulz-Ertner D Tsujii H . Particle Radiation Therapy Using Proton and Heavier Ion Beams. J Clin Oncol (2007) 25(8):953–64. doi: 10.1200/JCO.2006.09.7816 17350944

[12] Nomiya T Tsuji H Kawamura H Ohno T Toyama S Shioyama Y . A Multi-Institutional Analysis of Prospective Studies of Carbon Ion Radiotherapy for Prostate Cancer: A Report From the Japan Carbon Ion Radiation Oncology Study Group (J-CROS). Int J Radiat Oncol Biol Phys (2016) 97(1):91–7. doi: 10.1016/j.ijrobp.2016.09.019 27836119

[13] Slater JM Slater JD Kang JI Namihas IC Jabola BR Brown K . Hypofractionated Proton Therapy in Early Prostate Cancer: Results of a Phase I/II Trial at Loma Linda University. Int J Part (2019) 6(1):1–9. doi: 10.14338/IJPT-19-00057 PMC6871628 31773043

[14] Takagi M Demizu Y Fujii O Terashima K Niwa Y Daimon T . Proton Therapy for Localized Prostate Cancer: Long-Term Results From a Single-Center Experience. Int J Radiat Oncol Biol Phys (2020) 109(4):964–74. doi: 10.1016/j.ijrobp.2020.11.007 33186616

[15] Goetz G Mitic M Mittermayr T Wild C . Health Technology Assessment of Carbon-Ion Beam Radiotherapy: A Systematic Review of Clinical Effectiveness and Safety for 54 Oncological Indications in 12 Tumour Regions. Anticancer Res (2019) 39(4):1635–50. doi: 10.21873/anticanres.13269 30952702

[16] Takakusagi Y Katoh H Kano K Anno W Tsuchida K Mizoguchi N . Preliminary Result of Carbon-Ion Radiotherapy Using the Spot Scanning Method for Prostate Cancer. Radiat Oncol (2020) 15(1):127. doi: 10.1186/s13014-020-01575-7 32460889 PMC7254700

[17] Habl G Uhl M Katayama S Kessel KA Hatiboglu G Hadaschik B . Acute Toxicity and Quality of Life in Patients With Prostate Cancer Treated With Protons or Carbon Ions in a Prospective Randomized Phase II Study—The IPI Trial. Int J Radiat Oncol Biol Phys (2016) 95(1):435–43. doi: 10.1016/j.ijrobp.2016.02.025 27084659

[18] Arimura T Yoshiura T Matsukawa K Kondo N Kitano I Ogino T . Proton Beam Therapy Alone for Intermediate- or High-Risk Prostate Cancer: An Institutional Prospective Cohort Study. Cancers (2018) 10(4):116. doi: 10.3390/cancers10040116 PMC5923371 29642619

[19] Maruyama K Tsuji H Nomiya T Katoh H Ishikawa H Kamada T . Five-Year Quality of Life Assessment After Carbon Ion Radiotherapy for Prostate Cancer. J Radiat Res (2017) 58(2):260–6. doi: 10.1093/jrr/rrw122 PMC5439371 28043947

[20] Yang K Li X Bai Z . Research Methods of Evidence-Based Social Science: Systematic Review and Meta-Analysis. China: Lanzhou University Press (2018).

[21] Yang K . Evidence-Based Social Science: The Origin, Development and Prospects. Library Inf (2018) 2018(03):001–10. doi: 10.11968/tsyqb.1003-6938.2018038

[22] Wang XH Tian JH Zhang QN Li QR Zhang H Zhao L . Meta-Analysis of Carbon Ion Radiotherapy for Prostate Cancer. Chin J Radiol Med Prot (2016) 36(8):588–93. doi: 10.3760/cma.j.issn.0254-5098.2016.08.007

[23] Moher D Liberati A Tetzlaff J Altman DG . Preferred Reporting Items for Systematic Reviews and Meta-Analyses: The PRISMA Statement. Int J Surg (2010) 8(5):336–41. doi: 10.1016/j.ijsu.2010.02.007 20171303

[24] Stewart LA Tierney JF Clarke M . Cochrane Handbook for Systematic Reviews of Intervention. Cochrane (2011). Available at: www.training.cochrane.org/handbook.

[25] Car LT Li L Smith H Atun R . Cochrane Review: Search Strategies to Identify Observational Studies in MEDLINE and EMBASE. J Evid Based Med (2019) 12(3):225–6. doi: 10.1111/jebm.12358 31441237

[26] Higgins JPT Altman DG Sterne JAC . . Chapter 8: Assessing Risk of Bias in Included Studies[M]. Cochrane Handbook for Systematic Reviews of Interventions Version 5.1.0 [Updated March 2011]. In: The Cochrane Collaboration (2011). 187–214.

[27] Wells G Shea B O'Connell D Peterson J Welch V . Critical Evaluation of the Newcastle-Ottawa Scale for the Assessment of the Quality of Nonrandomized Studies in Meta-Analyses. Eur J Epidemiol (2010) 25:603–5. doi: 10.1007/s10654-010-9491-z 20652370

[28] Moga C Bing G Schopflocher D Harstall C . Development of a Quality Appraisal Tool for Case Series Studies Using a Modified Delphi Technique. (2012).

[29] Guyatt GH Oxman AD Schünemann HJ Tugwell P Knottnerus AJ . GRADE Guidelines: A New Series of Articles in the Journal of Clinical Epidemiology. J Clin Epidemiol (2011) 64(4):380–2. doi: 10.1016/j.jclinepi.2010.09.011 21185693

[30] Norris SL Meerpohl JJ Akl EA Schunemann HJ Gartlehner G Chen Y . The Skills and Experience of GRADE Methodologists Can be Assessed With a Simple Tool. J Clin Epidemiol (2016) 79:150–8. doi: 10.1016/j.jclinepi.2016.07.001 27421684

[31] Cao L Yang YJ Li ZW Wu HF Yang ZC Liu SX . Moderate Hypofractionated Radiotherapy Is More Effective and Safe for Localized Prostate Cancer Patients: A Meta-Analysis. Oncotarget (2016) 8(2):2647–58. doi: 10.18632/oncotarget PMC5356830 27926521

[32] Shimazaki J Tsuji H Ishikawa H Okada T Tsujii H . Carbon Ion Radiotherapy for Treatment of Prostate Cancer and Subsequent Outcomes After Biochemical Failure. Anticancer Res (2010) 30(12):5105–11. 21187497

[33] Nikoghosyan AV Schulz-Ertner D Herfarth K Didinger B Münter MW Jensen AD . Acute Toxicity of Combined Photon IMRT and Carbon Ion Boost for Intermediate-Risk Prostate Cancer – Acute Toxicity of 12C for PC. Acta Oncol (2011) 50(6):784–90. doi: 10.3109/0284186X.2011 21767175

[34] Ishikawa H Tsuji H Kamada T Akakura K Suzuki H Shimazaki J . Carbon-Ion Radiation Therapy for Prostate Cancer. Int J Urol (2012) 19(4):296–305. doi: 10.1111/j.442-2042.12.02961.x 22320843

[35] Okada T Tsuji H Kamada T Akakura K Suzuki H Shimazaki J . Carbon Ion Radiotherapy in Advanced Hypofractionated Regimens for Prostate Cancer: From 20 to 16 Fractions. Int J Radiat Oncol Biol Phys (2012) 84(4):968–72. doi: 10.1016/j.ijrobp.2012.01.072 22898380

[36] Katoh H Tsuji H Ishikawa H Kamada T Tsujii H . Health-Related Quality of Life After Carbon-Ion Radiotherapy for Prostate Cancer: A 3-Year Prospective Study. Int J Urol (2013) 21(4):370–5. doi: 10.1111/iju.12294 24118233

[37] Nomiya T Tsuji H Maruyama K Toyama S Suzuki H Akakura K . Phase I/II Trial of Definitive Carbon Ion Radiotherapy for Prostate Cancer: Evaluation of Shortening of Treatment Period to 3 Weeks. Br J Cancer (2014) 110(10):2389–95. doi: 10.1038/bjc.2014.191 PMC4021525 24722181

[38] Ishikawa H Katoh H Kaminuma T Kawamura H Nakano T . Carbon-Ion Radiotherapy for Prostate Cancer: Analysis of Morbidities and Change in Health-Related Quality of Life. Anticancer Res (2015) 35(10):5559–66. 26408726

[39] Zhang YF Li P Yu Q Wu S Chen X Zhang Q . Preliminary Exploration of Clinical Factors Affecting Acute Toxicity and Quality of Life After Carbon Ion Therapy for Prostate Cancer. Radiat Oncol (2019) 14(1):94. doi: 10.1186/s13014-019-1303-3 31164172 PMC6549341

[40] Kawamura H Kubo N Sato H Mizukami T Katoh H Ishikawa H . Moderately Hypofractionated Carbon Ion Radiotherapy for Prostate Cancer; a Prospective Observational Study “GUNMA0702”. BMC Cancer (2020) 20(1):75. doi: 10.1186/s12885-020-6570-8 32000716 PMC6990498

[41] Bryant C Smith TL Henderson RH Hoppe BS Mendenhall WM Nichols RC . Five-Year Biochemical Results, Toxicity, and Patient-Reported Quality of Life After Delivery of Dose-Escalated Image Guided Proton Therapy for Prostate Cancer. Int J Radiat Oncol Biol Phys (2016) 95(1):422–34. doi: 10.1016/j.ijrobp.2016.02.038 27084658

[42] Chuong MD Hartsell W Larson G Tsai H Laramore GE Rossi CJ . Minimal Toxicity After Proton Beam Therapy for Prostate and Pelvic Nodal Irradiation: Results From the Proton Collaborative Group REG001-09 Trial. Acta Oncol (2018) 57(3):368–74. doi: 10.1080/0284186X.2017 29034790

[43] Fang P Mick R Deville C Both S Bekelman JE Christodouleas JP . A Case-Matched Study of Toxicity Outcomes After Proton Therapy and Intensity-Modulated Radiation Therapy for Prostate Cancer. Cancer (2014) 121(7):1118–27. doi: 10.1002/cncr.29148 PMC4368478 25423899

[44] Henderson RH Bryant C Hoppe BS Nichols RC Mendenhall WM Flampouri S . Five-Year Outcomes From a Prospective Trial of Image-Guided Accelerated Hypofractionated Proton Therapy for Prostate Cancer. Acta Oncol (2017) 56(7):963–70. doi: 10.1080/0284186X.2017 28514929

[45] Henderson RH Hoppe BS Marcus RB Jr. Mendenhall WM Nichols RC Li Z . Urinary Functional Outcomes and Toxicity Five Years After Proton Therapy for Low- and Intermediate-Risk Prostate Cancer: Results of Two Prospective Trials. Acta Oncol (2013) 52(3):463–9. doi: 10.3109/0284186X.2013.764467 PMC3603169 23477359

[46] Ho CK Bryant CM Mendenhall NP Henderson RH Mendenhall WM Nichols RC . Long-Term Outcomes Following Proton Therapy for Prostate Cancer in Young Men With a Focus on Sexual Health. Acta Oncol (2018) 57(5):582–8. doi: 10.1080/0284186X.2018.1427886 29359988

[47] Iwata H Ishikawa H Takagi M Okimoto T Murayama S Akimoto T . Long-Term Outcomes of Proton Therapy for Prostate Cancer in Japan: A Multi-Institutional Survey of the Japanese Radiation Oncology Study Group. Cancer Med (2017) 7(3):677–89. doi: 10.1002/cam4.1350 PMC5852348 29441697

[48] Kim YJ Cho KH Pyo HR Lee KH Moon SH Kim TH . A Phase II Study of Hypofractionated Proton Therapy for Prostate Cancer. Acta Oncol (2013) 52(3):47–85. doi: 10.3109/0284186X.2013.764011 23398594

[49] Lee HJ Macomber MW Spraker MB Bowen SR Hippe D Fung A . Analysis of Gastrointestinal Toxicity in Patients Receiving Proton Beam Therapy for Prostate Cancer: A Single-Institution Experience. Adv Radiat Oncol (2019) 4(1):70–8. doi: 10.1016/j.adro.2018.08.002 PMC6349581 30706013

[50] Mendenhall NP Hoppe BS Nichols RC Mendenhall WM Morris CG Li Z . Five-Year Outcomes From 3 Prospective Trials of Image-Guided Proton Therapy for Prostate Cancer. Int J Radiat Oncol Biol Phys (2013) 88(3):596–602. doi: 10.1016/j.ijrobp.2013.11.007 24521677

[51] Mishra MV Khairnar R Bentzen SM Larson G Tsai H Sinesi C . Proton Beam Therapy Delivered Using Pencil Beam Scanning vs. Passive Scattering/Uniform Scanning for Localized Prostate Cancer: Comparative Toxicity Analysis of PCG 001-09. Clin Transl Radiat Oncol (2019) 19:80–6. doi: 10.1016/j.ctro.2019.08.006 PMC6804653 31650043

[52] Nakajima K Iwata H Ogino H Hattori Y Hashimoto S Nakanishi M . Acute Toxicity of Image-Guided Hypofractionated Proton Therapy for Localized Prostate Cancer. Int J Clin Oncol (2018) 23(2):353–60. doi: 10.1007/s10147-017-1209-8 29098520

[53] Nihei K Ogino T Onozawa M Murayama S Fuji H Murakami M . Multi-Institutional Phase II Study of Proton Beam Therapy for Organ-Confined Prostate Cancer Focusing on the Incidence of Late Rectal Toxicities. Int J Radiat Oncol Biol Phys (2011) 81(2):390–6. doi: 10.1016/j.ijrobp.2010.05.027 20832180

[54] Pan HY Jiang J Hoffman KE Tang C Choi SL Nguyen QN . Comparative Toxicities and Cost of Intensity-Modulated Radiotherapy, Proton Radiation, and Stereotactic Body Radiotherapy Among Younger Men With Prostate Cancer. J Clin Oncol (2018) 36(18):1823–30. doi: 10.1200/JCO.2017.75.5371 PMC6008105 29561693

[55] Santos PMG Barsky AR Hwang WT Deville C Wang X Both S . Comparative Toxicity Outcomes of Proton-Beam Therapy Versus Intensity-Modulated Radiotherapy for Prostate Cancer in the Postoperative Setting. Cancer (2019) 125(23):4278–93. doi: 10.1002/cncr.32457 PMC6856399 31503338

[56] Vargas CE Hartsell WF Dunn M Keole SR Doh L Chang J . Image-Guided Hypofractionated Proton Beam Therapy for Low-Risk Prostate Cancer: Analysis of Quality of Life and Toxicity, PCG GU 002. Rep Pract Oncol Radiother (2016) 21(3):207–12. doi: 10.1016/j.rpor.2016.01.002 PMC5002029 27601952

[57] Yu JB Soulos PR Herrin J Cramer LD Potosky AL Roberts KB . Proton Versus Intensity-Modulated Radiotherapy for Prostate Cancer: Patterns of Care and Early Toxicity. J Natl Cancer Inst (2012) 105(1):25–32. doi: 10.1093/jnci/djs463 23243199 PMC3536640

[58] Akakura K Tsujii H Morita S Tsuji H Yagishita T Isaka S . Phase I/II Clinical Trials of Carbon Ion Therapy for Prostate Cancer. Anal Bioanal Chem (2004) 379(2):188–91. doi: 10.1007/s00216-003-2479-8 14743464

[59] Ishikawa H Tsuji H Kamada T Yanagi T Mizoe JE Kanai T . Carbon Ion Radiation Therapy for Prostate Cancer: Results of a Prospective Phase II Study. Clin Oncol (R Coll Radiol) (2006) 18(7):577–8. doi: 10.1016/j.clon.2006.05.011 16971008

[60] Tsujii H . Clinical Results of Carbon Ion Radiotherapy at NIRS. J Radiat Res (2007) 48(Suppl.A):A1–A13. doi: 10.1269/jrr.48.a1 17513896

[61] Hanks GE . Conformal Radiotherapy for Prostate Cancer. Ann Med (2000) 32(1):57–63. doi: 10.3109/07853890008995911 10711579

[62] Pollack A Zagars GK Starkschall G Antolak JA Lee JJ Huang E . Prostate Cancer Radiation Dose Response: Results of the M. D. Anderson Phase III Randomized Trial. Int J Radiat Oncol Biol Phys (2002) 53(5):1097–105. doi: 10.1016/s0360-3016(02)02829-8 12128107

[63] Kupelian PA Willoughby TR Reddy CA Klein EA Mahadevan A . Hypofractionated Intensity-Modulated Radiotherapy (70 Gy at 2.5 Gy Per Fraction) for Localized Prostate Cancer: Cleveland Clinic Experience. Int J Radiat Oncol Biol Phys (2007) 68(5):1424–30. doi: 10.1016/j.ijrobp.2007.01.067 17544601

[64] Sveistrup J af Rosenschöld PM Deasy JO Oh JH Pommer T Petersen PM . Improvement in Toxicity in High Risk Prostate Cancer Patients Treated With Image-Guided Intensity-Modulated Radiotherapy Compared to 3D Conformal Radiotherapy Without Daily Image Guidance. Radiat Oncol (2014) 9:44. doi: 10.1186/1748-717X-9-44 24495815 PMC3922544

[65] Xiu-xia L Ya Z Yao-long C Ke-hu Y Zong-jiu Z . The Reporting Characteristics and Methodological Quality of Cochrane Reviews About Health Policy Research. Health Policy (2015) 119(4):503–10. doi: 10.1016/j.healthpol.2014.09.002 25260911

[66] Yao L Sun R Chen YL Wang Q Yang K . The Quality of Evidence in Chinese Meta-Analyses Needs to be Improved. J Clin Epidemiol (2016) 74:73–9. doi: 10.1016/j.jclinepi.2016.01.003 26780259

[67] Li Y Cao L Zhang Z Hou L Qin Y Hui X . Reporting and Methodological Quality of COVID-19 Systematic Reviews Needs to be Improved: An Evidence Mapping. J Clin Epidemiol (2021) 135:17–28. doi: 10.1016/j.jclinepi.2021.02.021 33657455 PMC8313077

[68] Tian J Zhang J Ge L Yang K Song F . The Methodological and Reporting Quality of Systematic Reviews From China and the USA Are Similar. J Clin Epidemiol (2017) 85:50–8. doi: 10.1016/j.jclinepi.2016.12.004 28063911

[69] Yan P Yao L Li H Zhang M Xun Y Li M . The Methodological Quality of Robotic Surgical Meta-Analyses Needed to be Improved: A Cross-Sectional Study. J Clin Epidemiol (2019) 109:20–9. doi: 10.1016/j.jclinepi.2018.12.013 30579979

[70] Yao X Florez ID Zhang P Zhang C Zhang Y Wang C . Clinical Research Methods for Treatment, Diagnosis, Prognosis, Etiology, Screening, and Prevention: A Narrative Review. J Evid Based Med (2020) 13(2):130–6. doi: 10.1111/jebm.12384 32445266

