# Supplementary material for: Clinical Efficacy and Safety of Proton and Carbon Ion Radiotherapy for Prostate Cancer: A Systematic Review and Meta-Analysis
Source: Front Oncol. 2021 Oct 12;11:709530. doi: 10.3389/fonc.2021.709530 (PMC8547329; doi:10.3389/fonc.2021.709530)
Supplement: Supplementary file 1 [file DataSheet_1.docx]

Supplementary Material

# Search strategy

## PubMed (from 1 January 2010 to 13 July 2021)

| **Search number** | **Query** | **Results** |
| --- | --- | --- |
| #1 | "Heavy Ions"[Mesh] | 1,142 |
| #2 | "Heavy Ion Radiotherapy"[Mesh] | 5,141 |
| #3 | "Protons"[Mesh] | 29,986 |
| #4 | (((((heavy ion*[Title/Abstract]) OR (Carbon ion*[Title/Abstract])) OR (C-ion*[Title/Abstract])) OR (Particle[Title/Abstract])) OR (Ion[Title/Abstract])) OR (Proton*[Title/Abstract]) | 627,821 |
| #5 | #1 OR #2 OR #3 OR #4 | 635,015 |
| #6 | "Prostatic Neoplasms"[Mesh] | 134,615 |
| #7 | (((((Prostate Neoplasm*[Title/Abstract]) OR (Prostatic Neoplasm*[Title/Abstract])) OR (Prostate Cancer*[Title/Abstract])) OR (Prostatic Cancer*[Title/Abstract])) OR (Prostate Tumor*[Title/Abstract])) OR (Prostatic Tumor*[Title/Abstract]) | 135,824 |
| #8 | #6 OR #7 | 169,923 |
| #9 | #5 AND 38 | 2,049 |
| #10 | #9 AND ((humans[Filter]) AND (2010/1/1:2021/7/13[pdat])) | 1108 |

## EMBASE (from 1 January 2010 to July 2021)

| **Search number** | **Query** | **Results** |
| --- | --- | --- |
| #1 | 'heavy ion'/exp | 1,557 |
| #2 | 'ion therapy'/exp | 2,478 |
| #3 | 'proton'/exp | 46,170 |
| #4 | 'heavy ion*':ab,ti OR 'carbon ion*':ab,ti OR 'c ion*':ab,ti OR particle:ab,ti OR ion:ab,ti OR proton*:ab,ti | 683,781 |
| #5 | #1 OR #2 OR #3 OR #4 | 693,856 |
| #6 | 'prostate tumor'/exp | 264,393 |
| #7 | 'prostate neoplasm*':ab,ti OR 'prostatic neoplasm*':ab,ti OR 'prostate cancer*':ab,ti OR 'prostatic cancer*':ab,ti OR 'prostate tumor*':ab,ti OR 'prostatic tumor*':ab,ti | 198,848 |
| #8 | #6 OR #7 | 280,357 |
| #9 | #5 AND #8 | 3,416 |
| #10 | #9 AND 'human'/de | 2,898 |
| #11 | #9 AND 'human'/de AND [embase]/lim NOT ([embase]/lim AND [medline]/lim) | 1,088 |

## Cochrane library (from 1 January 2010 to July 2021)

| **Search number** | **Query** | **Results** |
| --- | --- | --- |
| #1 | MeSH descriptor: [Heavy Ion Radiotherapy] explode all trees | 50 |
| #2 | MeSH descriptor: [Heavy Ions] explode all trees | 1 |
| #3 | MeSH descriptor: [Protons] explode all trees | 109 |
| #4 | (heavy ion*):ti,ab,kw OR (Carbon ion*):ti,ab,kw OR (C-ion*):ti,ab,kw OR (Particle):ti,ab,kw OR (Ion):ti,ab,kw | 11940 |
| #5 | (Proton*):ti,ab,kw (Word variations have been searched) | 5993 |
| #6 | # 240 OR #241 OR #242 OR #243 OR #244 | 34901 |
| #7 | MeSH descriptor: [Prostatic Neoplasms] explode all trees | 5746 |
| #8 | (Prostate Neoplasm*):ti,ab,kw OR (Prostatic Neoplasm*):ti,ab,kw OR (Prostate Cancer*):ti,ab,kw OR (Prostatic Cancer*):ti,ab,kw OR (Prostate Tumor*):ti,ab,kw | 15237 |
| #9 | (Prostatic Tumor*):ti,ab,kw | 3334 |
| #10 | #246 or #247 or #248 | 15237 |
| #11 | #245 AND #249 | 424 |

## Web of science (from 1 January 2010 to July 2021)

| **Search number** | **Query** | **Results** |
| --- | --- | --- |
| #1 | TS=(heavy ion* ) OR TS=(Carbon ion*) OR TS=(C-ion*) OR TS=(Particle) OR TS=(Ion) OR TS=(Proton*) Index Date: 2010-01-01 to 2021-07-13 | 1,753,023 |
| #2 | TS=(Prostate Neoplasm*) OR TS=(Prostatic Neoplasm*) OR TS=(Prostate Cancer*) OR TS=( Prostatic Cancer*) OR TS=(Prostate Tumor*) OR TS=(Prostatic Tumor*) Index Date: 2010-01-01 to 2021-07-13 | 158,054 |
| #3 | #1 AND #2 | 3,758 |

# Risk of bias assessment (cohort studies)

| **Study, year** | **Selection** | | | | **Comparability** | | **Outcome** | | | |  | |
| --- | --- | --- | --- | --- | --- | --- | --- | --- | --- | --- | --- | --- |
|  | Representativeness of the exposed cohort | Selection of the non exposed cohort | Ascertainment of exposure | Demonstration that outcome of interest was not present at start of study | Comparability of cases and controls on the basis of the design or analysis | Ascertainment of outcome | | Was follow-up long enough for outcomes to occur | Adequacy of follow up of cohorts | **Quality score** | |  |
| Nakajima 2018 | * | * | * | * | / | * | | / | * | 7 | |  |
| Fang 2014 | * | * | * | * | ** | * | | * | * | 9 | |  |
| Pan 2018 | * | * | * | * | ** | * | | / | / | 7 | |  |
| Santos 2019 | * | * | * | * | / | * | | * | * | 8 | |  |
| Yu  2012 | * | * | * | * | * | * | | / | * | 8 | |  |
| Mishra 2019 | * | * | * | * | / | * | | * | * | 8 | |  |

# Forest plots of outcomes


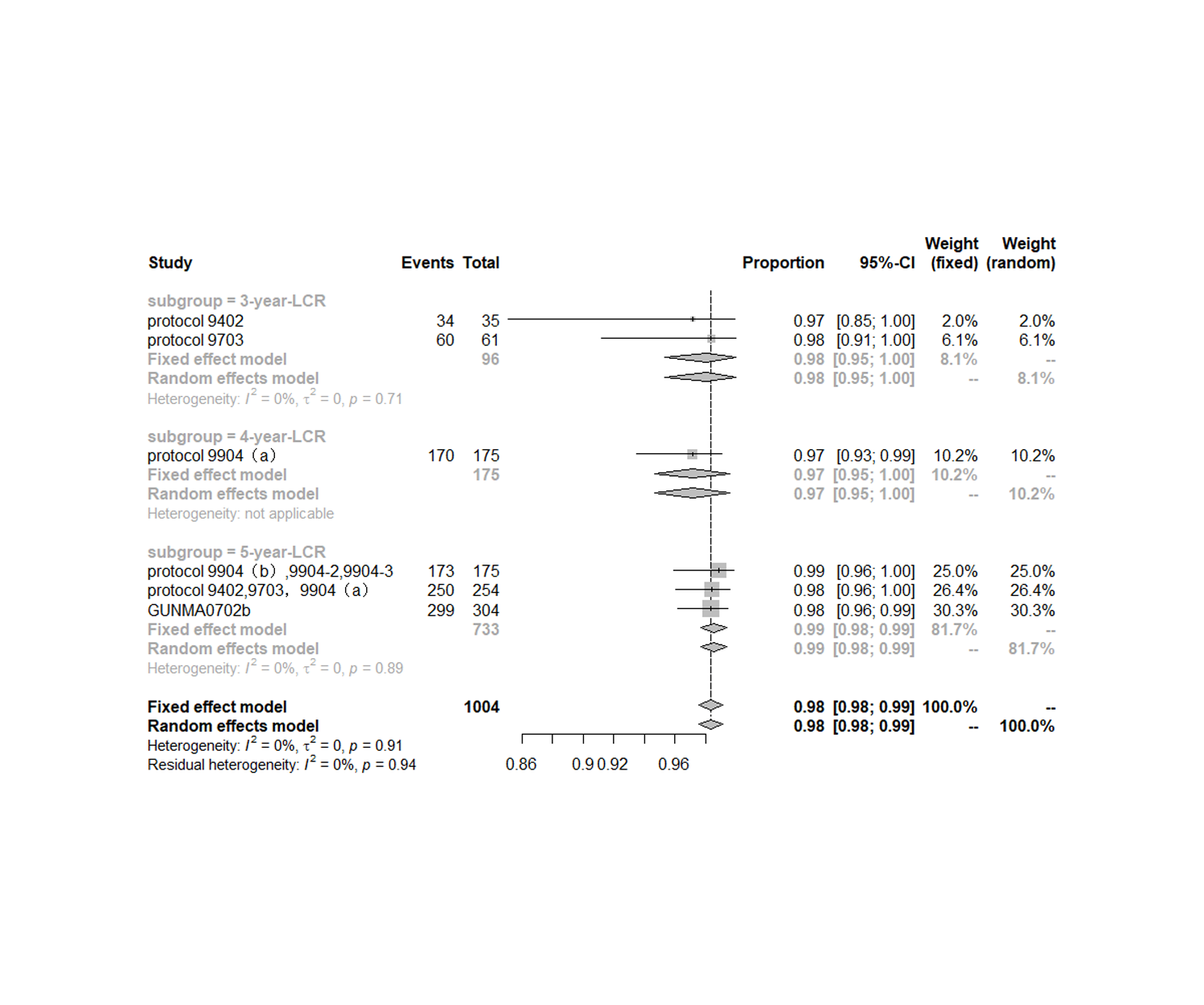


**FIGURE 1 Forest plots of the LCR of CIRT for prostate cancer**


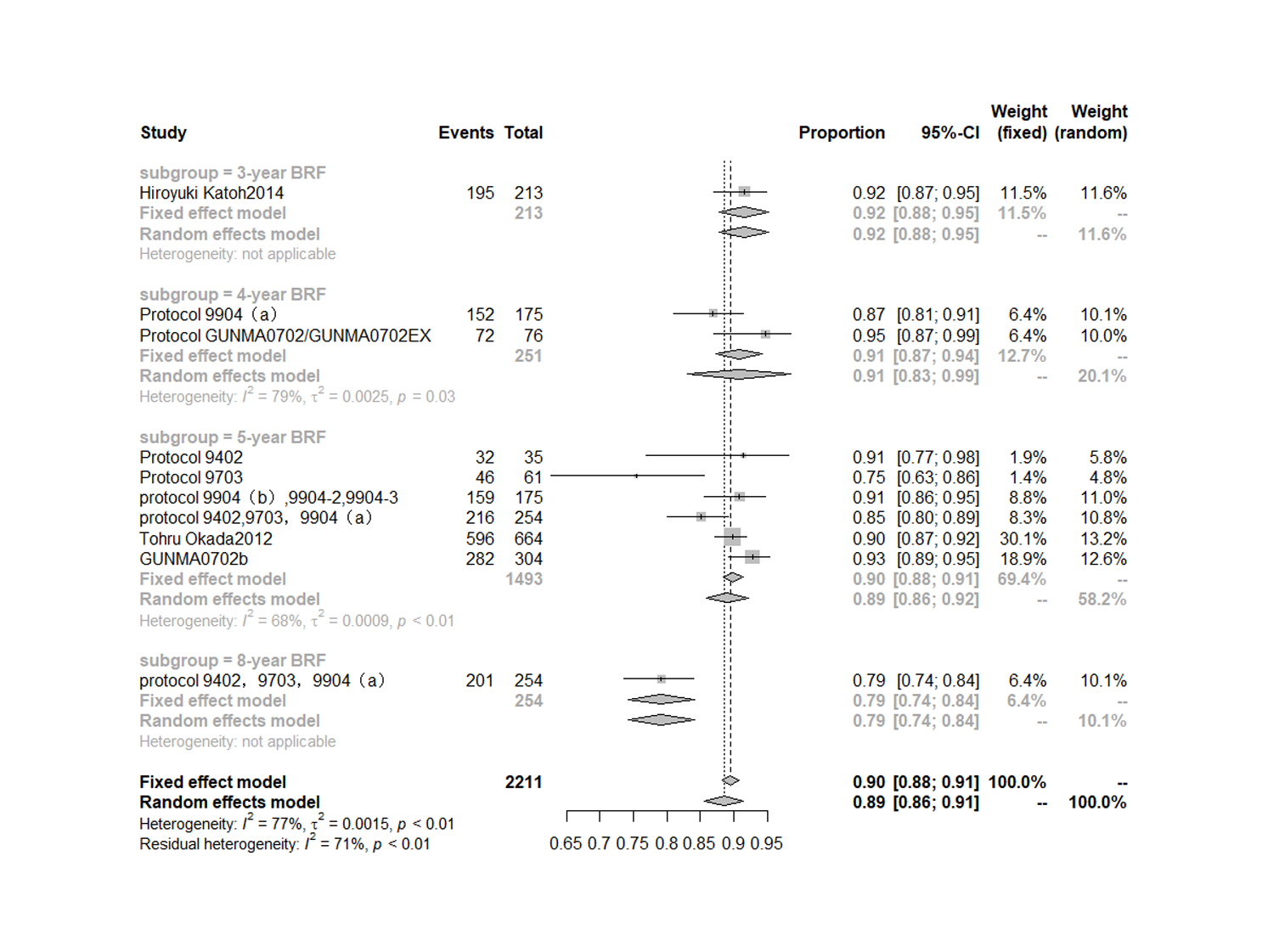


**FIGURE 2 Forest plots of the BRF of CIRT for prostate cancer**


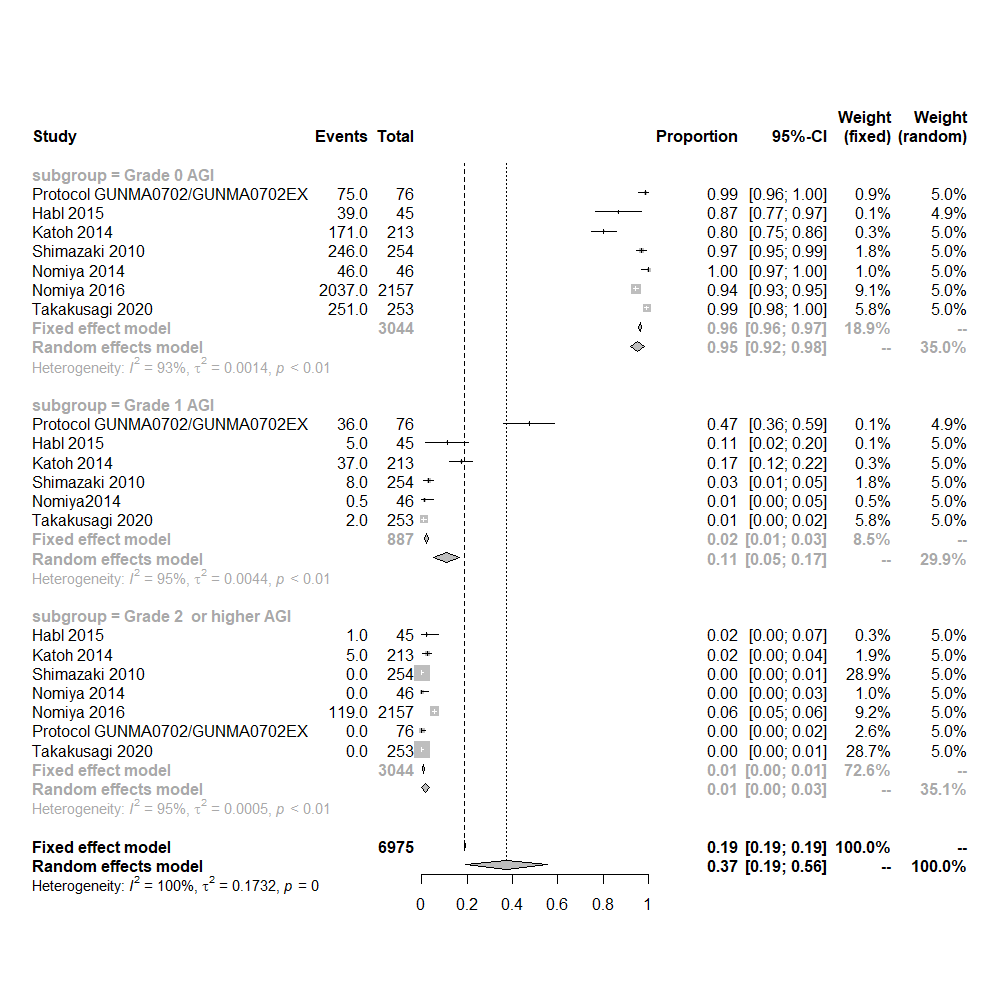


**FIGURE 3 Forest plots of the AGI of CIRT for prostate cancer**


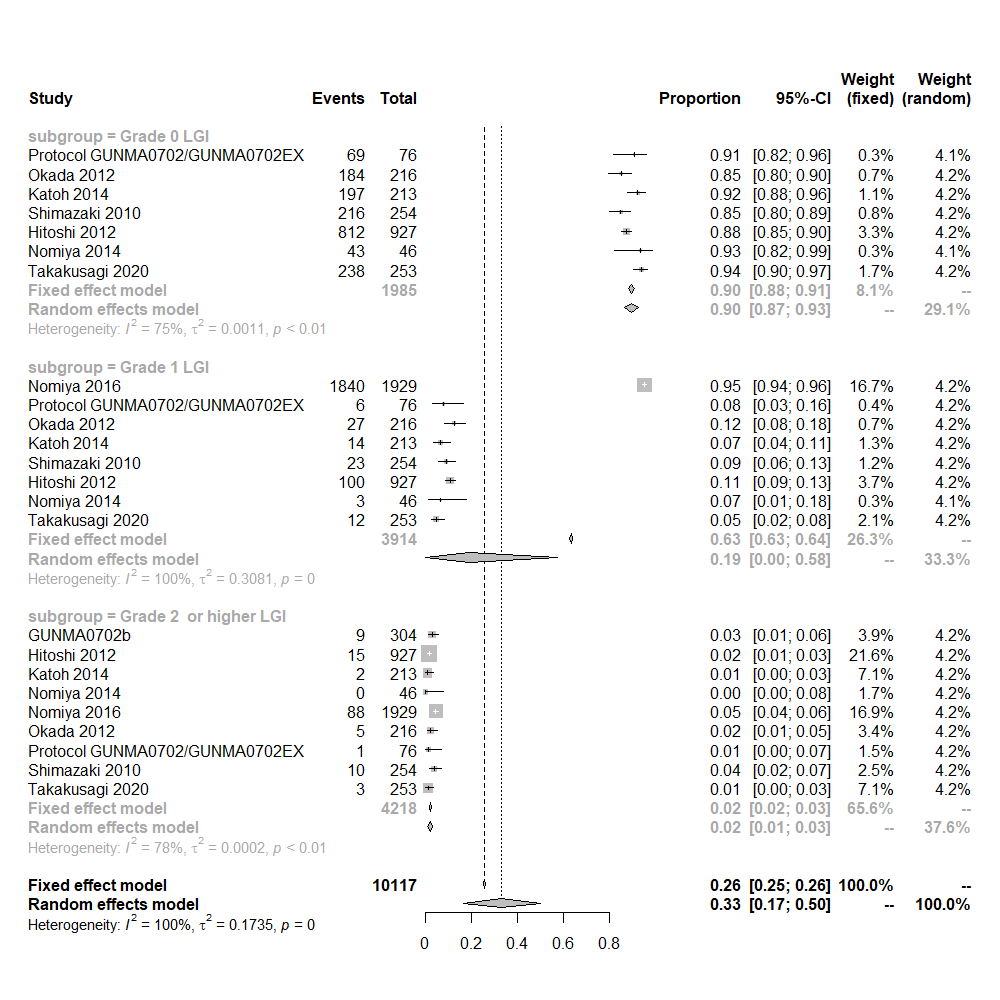


**FIGURE 4 Forest plots of the LGI of CIRT for prostate cancer**


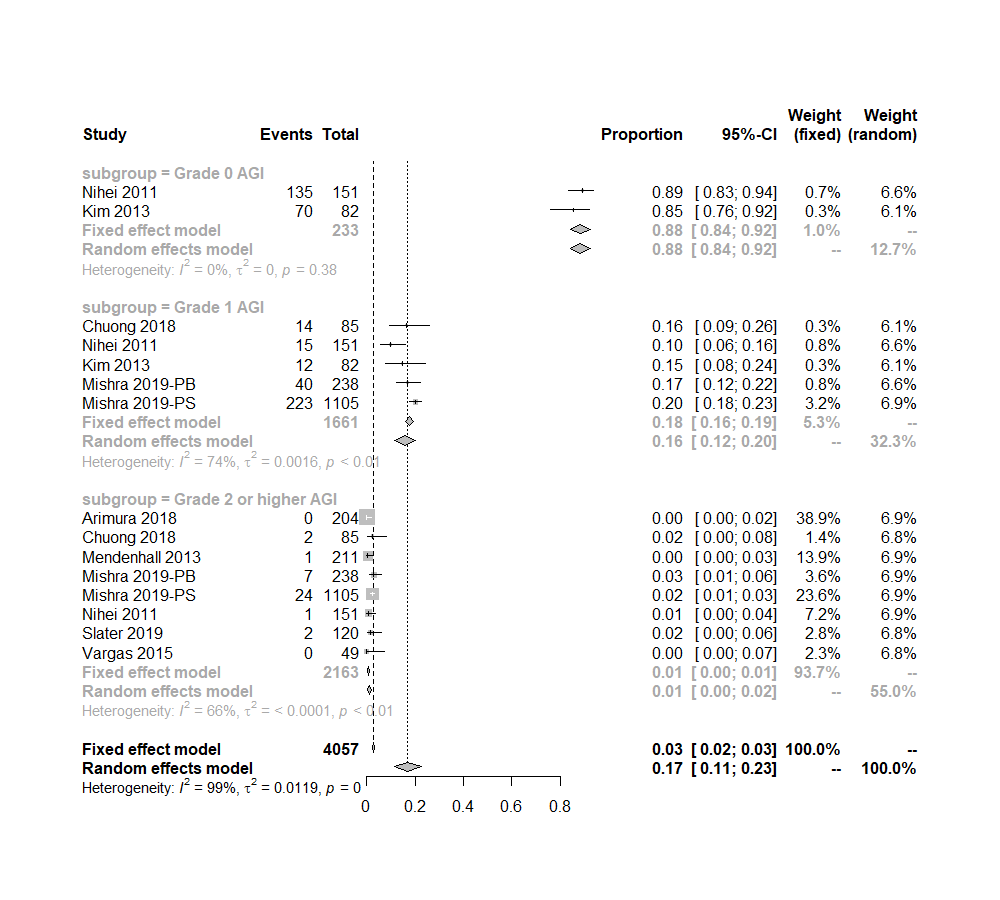


**FIGURE 5 Forest plots of the AGI of PBT for prostate cancer**


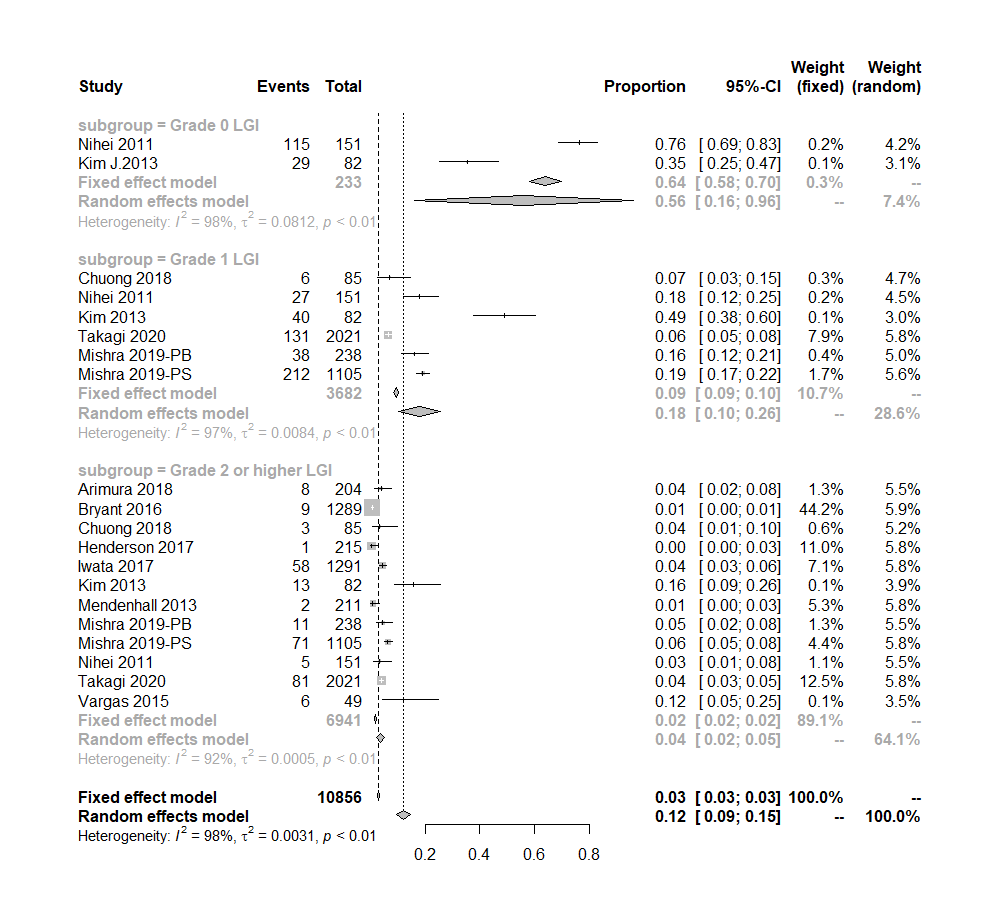


**FIGURE 6 Forest plots of the lGI of PBT for prostate cancer**


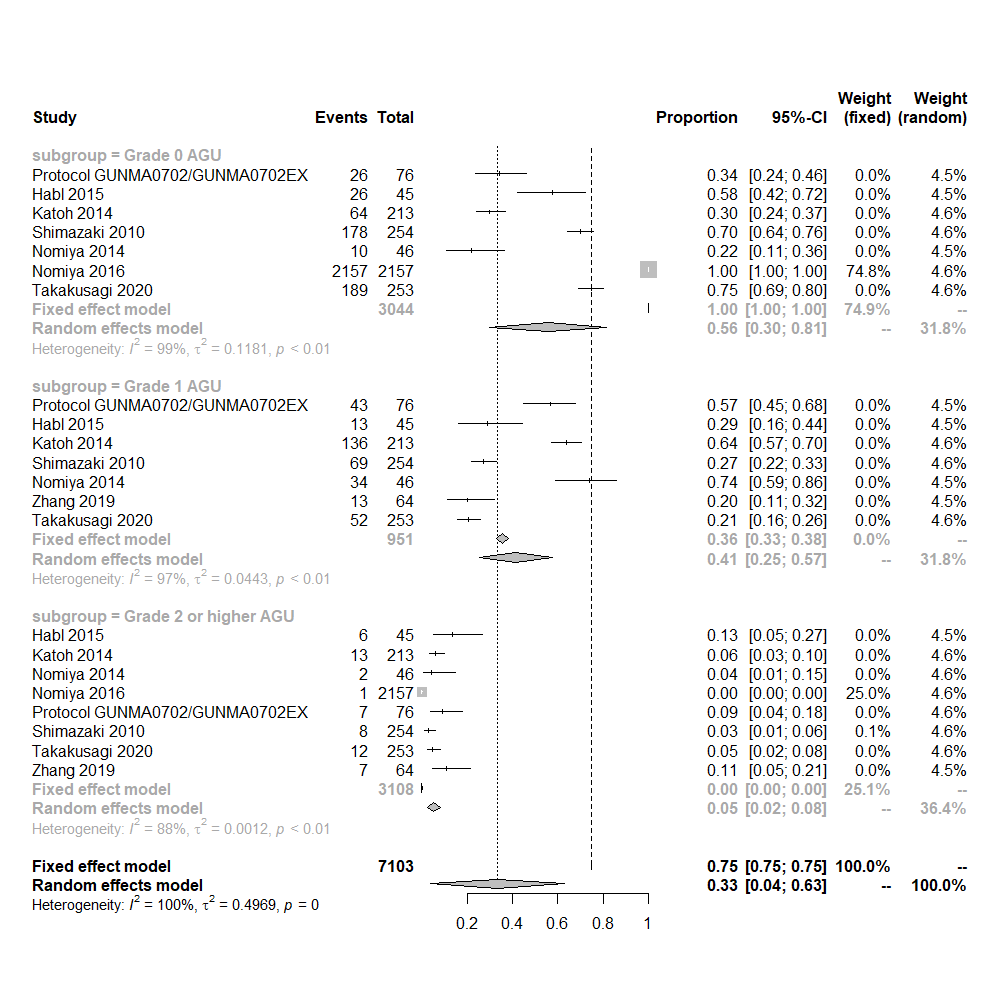
**FIGURE 7 Forest plots of the AGU of CIRT for prostate cancer**


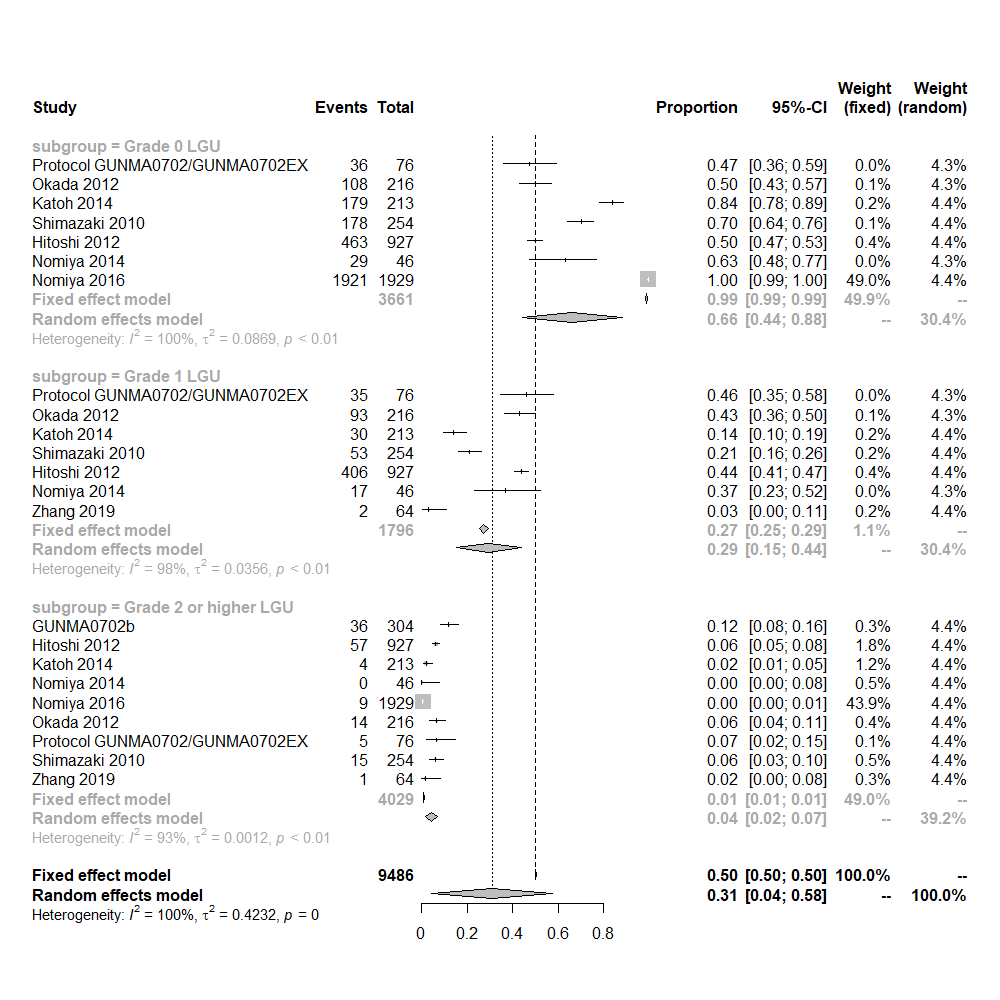


**FIGURE 8 Forest plots of the LGU of CIRT for prostate cancer**


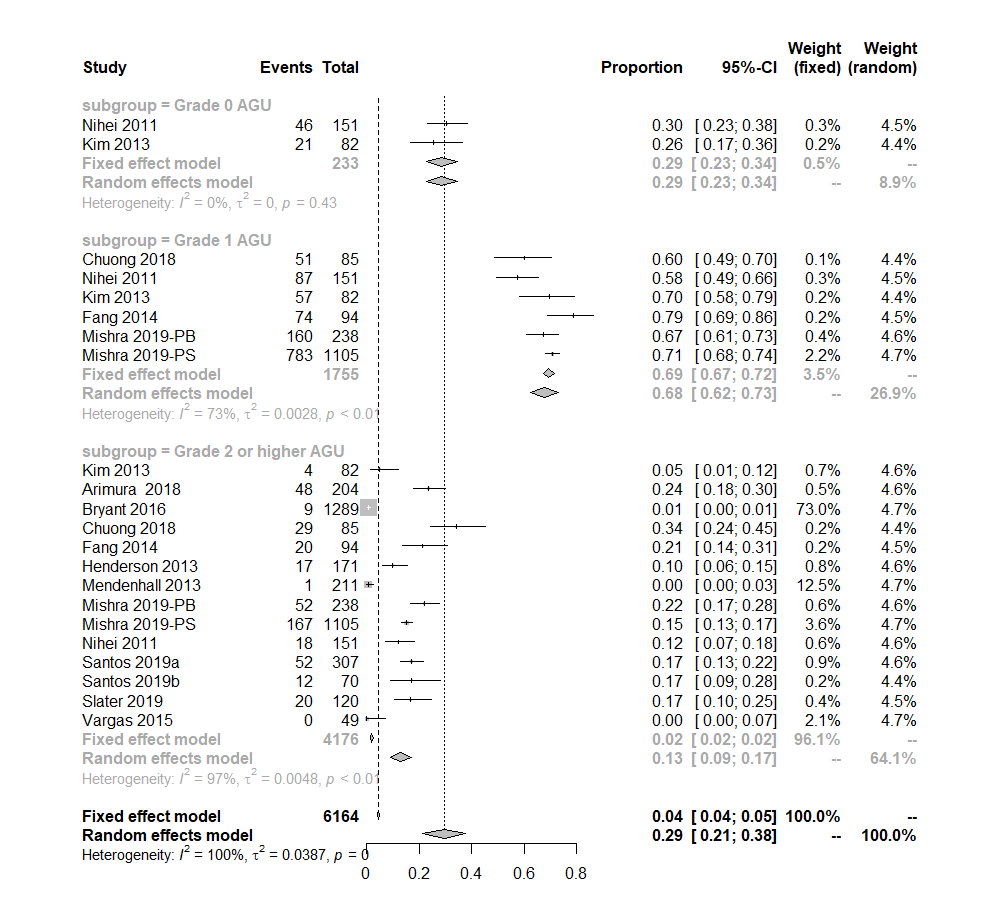


**FIGURE 9 Forest plots of the AGU of PBT for prostate cancer**


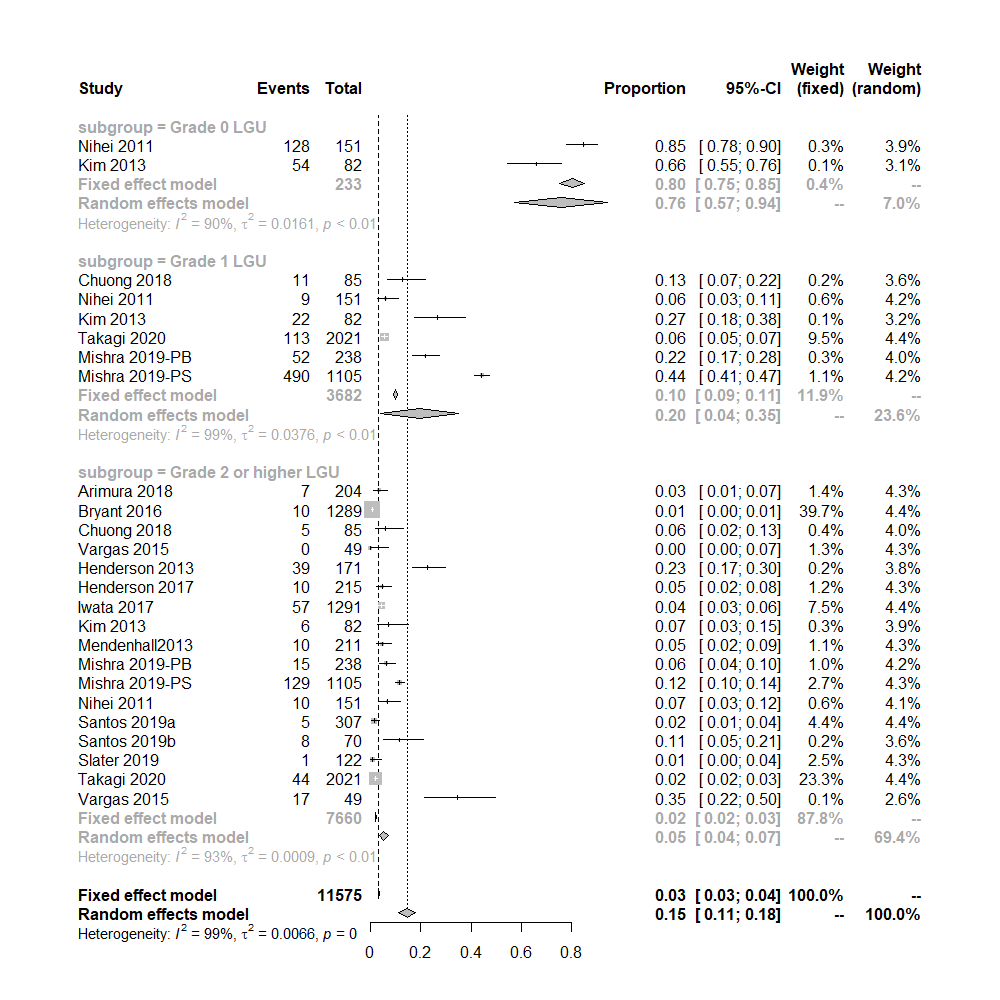


**FIGURE 10 Forest plots of the LGU of PBT for prostate cancer**


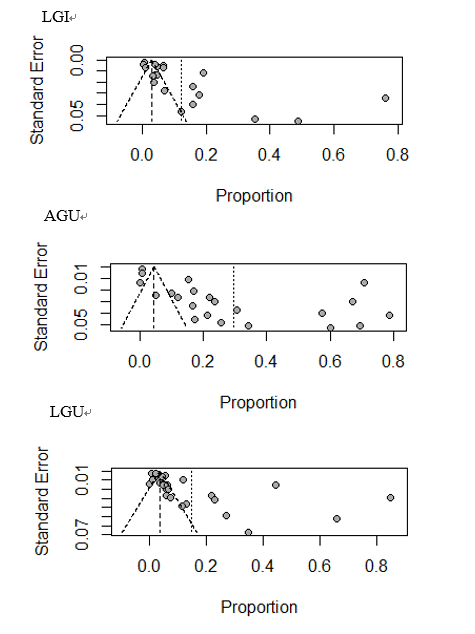


**FIGURE 11 Funnel plots of the LGI, AGU and LGU of PBT for prostate cancer**
